# A Machine-learning Approach to Forecast Aggravation Risk in Patients with Acute Exacerbation of Chronic Obstructive Pulmonary Disease with Clinical Indicators

**DOI:** 10.1038/s41598-020-60042-1

**Published:** 2020-02-20

**Authors:** Junfeng Peng, Chuan Chen, Mi Zhou, Xiaohua Xie, Yuqi Zhou, Ching-Hsing Luo

**Affiliations:** 1 Sun Yat-sen University, School of Data and Computer Science, Guangzhou, 510006 China; 2 Sun Yat-sen University, The Third Affiliated Hospital, Guangzhou, 510640 China

**Keywords:** Prognostic markers, Chronic obstructive pulmonary disease

## Abstract

Patients with chronic obstructive pulmonary disease (COPD) repeat acute exacerbations (AE). Global Initiative for Chronic Obstructive Lung Disease (GOLD) is only available for patients in stable phase. Currently, there is a lack of assessment and prediction methods for acute exacerbation of chronic obstructive pulmonary disease (AECOPD) patients during hospitalization. To enhance the monitoring and treatment of AECOPD patients, we develop a novel C5.0 decision tree classifier to predict the prognosis of AECOPD hospitalized patients with objective clinical indicators. The medical records of 410 hospitalized AECOPD patients are collected and 28 features including vital signs, medical history, comorbidities and various inflammatory indicators are selected. The overall accuracy of the proposed C5.0 decision tree classifier is 80.3% (65 out of 81 participants) with 95% Confidence Interval (CI):(0.6991, 0.8827) and Kappa 0.6054. In addition, the performance of the model constructed by C5.0 exceeds the C4.5, classification and regression tree (CART) model and the iterative dichotomiser 3 (ID3) model. The C5.0 decision tree classifier helps respiratory physicians to assess the severity of the patient early, thereby guiding the treatment strategy and improving the prognosis of patients.

## Introduction

Chronic obstructive pulmonary disease (COPD) as a major cause of chronic morbidity and mortality is a global health threat, and will become the third leading cause of death in the world by 2030^1^. In 2012, 3 million deaths caused by COPD, and the amount is equivalent to 6% of the total death that year^2^. COPD is characterized by chronic bronchitis, chronic airway obstruction, and emphysema, which causes progressive, irreversible decline in lung function^3^. COPD patients with acute exacerbation need to be admitted to the hospital with a mortality rate of approximately 10%^4^. Inflammatory cells infiltrate the small airways, releasing destructive enzymes and inflammatory factors, leading to damage and remodeling of small airways^5^. Neutrophils are the main inflammatory cells in patients with COPD, which release neutrophil elastase(NE) and myeloperoxidase(MPO)^6^. Macrophages are another major inflammatory cell that produces TNF-*α*, IL-8,reactive oxygen species(ROS), and matrix metalloproteinases(MMP)^7^. Eosinophils and lymphocytes are also widely present in the airways of patients with COPD, but their mechanisms of damage to the airways are still controversial^8^.

However, early intervention on these patients with COPD can reduce morbidity and mortality^9,10^. The socio-economic burden caused by the deterioration of COPD cannot be underestimated^11^. Global Initiative for Chronic Obstructive Lung Disease (GOLD) has been employed to classify the severity of COPD patients since 2011^12^. However, GOLD is unable to fully guide the clinical treatment of COPD patients due to the complexity of the progression of COPD, exacerbation recovery time, risk of re-admission and intensive care unit (ICU) admission^13–15^.

In order to accurately classify the severity of COPD patients, a lot of scholars have investigated the machine learning algorithms to assist clinical decision-making. Nunavath *et al.* utilized feed-forward neural networks (FFNN) for the classification of COPD patients category and Long Short-Term Memory (LSTM), for early prediction of COPD exacerbations and subsequent triage. However, the follow-up patients’ data collected from the family environment are prone to be interfered by multiple factors, leading to the deterioration of data quality^16^. Tang *et al*. developed a four-layer deep learning model that utilizes a specially configured recurrent neural network to handling temporal variation in COPD progression. The complexity of the proposed model led to poor interpretation^17^. Almagro *et al*. took advantage of the charlson index and a questionnaire to study the comorbidities and short-term prognosis in patients hospitalized for acute exacerbation of COPD. The study did not consider the role of inflammation in the prognosis of COPD^18^. Thomsen *et al*. foud that the C-Reactive Protein (CRP), fibrinogen and leukocyte count were important inflammatory biomarkers which were associated with increased risk of exacerbations of COPD. Nevertheless, the study had the problem of unbalanced samples, such as too few non-viable samples, which may affect the reliability of the experimental conclusion^19^. Karadeniz *et al*. discovered that increased mortality risk was found to be associated with high CRP values. But the population of COPD in the study was too small^20^.

Further, a considerable number of studies have shown that inflammatory cells and their secretions in the lungs are closely related to the severity and acute exacerbation of COPD^21,22^. However, most of these studies examined induced sputum and bronchoalveolar lavage fluid, which is difficult to apply to clinical use on a large scale. In addition to pulmonary inflammation, COPD patients also have systemic inflammation^23^. The detection of serum inflammatory markers is widely used in clinical practice, and the correlation between inflammatory markers and the severity and prognosis of patients with COPD is concerned. Some studies have shown that levels of inflammatory markers such as CRP and PCT are related to the severity of COPD patients and can guide the application of anti-infective therapy^24,25^.

It is of great significance to establish a more generalized and explanatory model to classify the severity of AECOPD patients with more clinical indicators. The C5.0 decision tree is a classification approach which generates the tree in top-down scheme based on the information using a recursive process^26,27^. The core idea of decision tree is to construct an optimized tree to distinguish the positive and negative samples^28^. C5.0 is employed in medical decision making as it can simultaneously provide high classification accuracy and simple representation of the clinic data^29^. Lakshmi *et al*. investigated the decision tree as a supervised classification method in the medical diagnoses^30^. C5.0 is an improved version of well-known and widely used C4.5 algorithm, proposed by Ross Quinlan^31,32^. The C5.0 algorithm is highly interpretable and is more accurate, much faster and needs less memory than C4.5 and classification and regression tree (CART). C5.0 as a boosting algorithm achieves a much better generality ability than a single learner, such as C4.5, CART and ID3. Boosting are also considered the most popular and effective approaches to improve the generality ability of the single model^33,34^. Further, boosting tends to produce a more diverse set of learners than bagging does^35^.

To build a generic machine model to support the diagnosis of the deterioration and death risk of AECOPD for physicians, we develop a C5.0 decision tree classifier using basic indicators, comorbidity, and inflammatory indicators. The prediction result shows that C5.0 classifier is a promising research tool to help the physicians to obtain the severity assessment of the patients after admission.

## Results

A confusion matrix is a contingency table which is widely used to represent the classification results, typically a supervised learning^36^. It allows the visualization of the performance of the classification algorithm. Each column of the confusion matrix denotes the actual classes while each row denotes the predicted class (or vice versa)^37^. Precision, recall, F1-measure and overall accuracy are the metrics which are employed to evaluate our C5.0 classifier based on True Positives (TP), False Positives (FP), True Negative (TN) and False Negatives (FN). Metrics are defined as follows:

1 $$Precision=\frac{TP}{TP+FP},$$

2 $$Recall=\frac{TP}{TP+FN}.$$

3 $$F-Measure=2\times \frac{Precision\times Recall}{Precision+Recall},$$

4 $$Accuracy=\frac{TP+TN}{TP+FN+FT+FN}.$$

**Figure 1:**
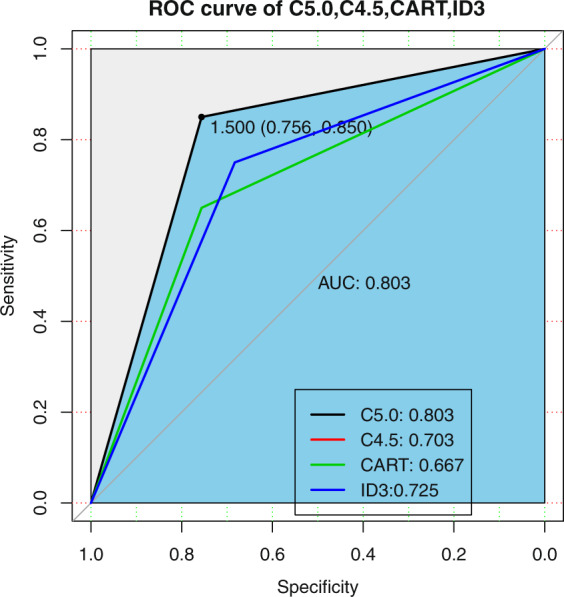
The receiver operating characteristic of the proposed C5.0 classifier compared with boosting C45, CART and ID3.

The overall accuracy of the proposed C5.0 classifier consisting of 40 independent decision trees is 80.3% (329 out of 410 participants) with 95% Confidence Interval (CI):(0.6991, 0.8827) and Kappa 0.6054. The detailed evaluation of the C5.0 classifier is shown in Table 1. The receiver operating characteristic (ROC) of C5.0, CART, ID3 algorithm are shown in Fig. 1. The area under curve (AUC) of C5.0, CART and ID3 are 0.803, 0.654 and 0.725 respectively. AUC can accurately reflect the relationship between specificity and sensitivity of an analytical method and is a measure of the accuracy of the validation.

**Table 1:** Evaluation of the C5.0 classifier voted by the 40 independent decision trees.

	Precision	Recall	F-Measure
Mild	85.7%	73.2%	0.7895
Severe	76.1%	87.5%	0.8140
The overall accuracy is 80.3%

The proposed C5.0 enables a boosting procedure to build 40 decision trees (Table 2). We exploit the global pruning step to simplify the trees. The average tree size of the 40 decision trees generated by C5.0 is 29.8. Early stopping approach is used to build a C5.0 model with the strong generalization ability. The number of average misclassification and misclassification rate of the 40 decision trees was 54.8 and 16.7% before the weighted voting.

**Table 2:** Summary of the C5.0 classifier constructed by 40 independent decision trees.

Number of decision trees	Average tree size	Average misclassification number	Average misclassification rate
40	29.8	54.8	16.7%

**Figure 2:**
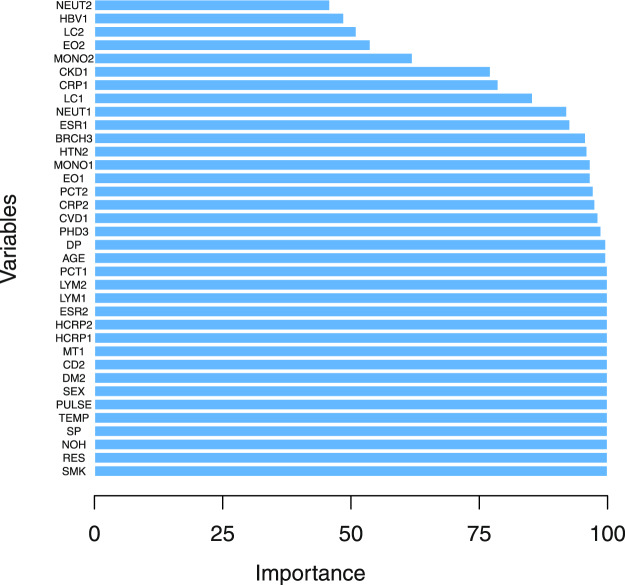
The variables importance of the proposed C5.0 classifier. Note that SMK, AGE, NOH, TEMP, PULSE, RES, SP, DP, PHD, BRCH, HTN, DM, CHD, CKD, MT, CD, HBV and CRHS denote smoking history, age, number of hospitalizations, temperature, pulse rate, respiratory rate, systolic pressure, diastolic pressure, pulmonary heart disease, bronchiectasis, yypertension, diabetes mellitus, coronary heart disease, chronic kidney disease, malignant tumor, cerebrovascular disease, viral hepatitis and Cirrhosis, respectively. While CRP, EO, ESR, HCRP,LC, LYM, MONO, NEUT and PCT are C-reactive protein, absolute value of eosinophils, erythrocyte sedimentation rate, high sensitivity c-reactive protein, leukocyte count, lymphocyte absolute value, nuclear cell absolute value, absolute neutrophils and detection of procalcitonin, respectively.

We also calculates the variable importance (attribute usage) for the C5.0 models (Fig. 2). Figure 2 shows that basic indicators (smoking history, age, respiratory rate, number of hospitalizations and temperature), comorbidities (pulmonary heart disease,bronchiectasis,cerebrovascular disease and coronary heart disease) and inflammation (C-reactive protein, high sensitivity c-reactive protein, erythrocyte sedimentation rate, leukocyte count, absolute neutrophils and lymphocyte absolute value) are the most predictive indicators. The variable importance is in line with clinical observation basically. These indicators may act as key indicators for the aggravation risk prediction for the AECOPD patients in clinical diagnosis.

In order to verify the stability of the model, we calculated the predictive performance of the model at different division choices of training/testing samples (Table 3). We considered five sample division ratios (90%/10%, 80%/20%, 70%/30%, 60%/40% and 50%/50%) between training set and testing set. We found that C5.0 achieved the best predictive classification performance at different different division choices. Figure 2 showed that the variables importance of the C5.0 model.

**Table 3:** Sensitivity analysis on different choices of training/testing samples (Accuracy).

Method	Sample division ratio between training set (%) and testing set (%)					
	90/10	80/20	70/30	60/40	50/50	Mean ± STD
**C5.0**	**0.725**	**0.803**	**0.730**	**0.724**	**0.702**	**0.737** ± **0.039**
ABC4.5	0.65	0.704	0.713	0.699	0.629	0.679 ± 0.037
ABCART	0.675	0.667	0.639	0.712	0.654	0.669 ± 0.027
ABID3	0.575	0.728	0.631	0.644	0.624	0.64 ± 0.0555

We can find that the proposed C5.0 classifier performs better than the CART and ID3, which is able to describe the intrinsic nature of datasets and to be of a significant value in a variety of applications involving data classification. This C5.0 decision tree model for early detection could trigger the initiation of timely treatment, thereby potentially reducing exacerbation severity and recovery time and improving the patient’s health.

## Discussion

In this work, we use C5.0 decision classifier to fast identify the deterioration and death risk of AECOPD patients using basic indicators, comorbidities, and inflammation after admission. The C5.0 decision classifier proposed in this paper predict 80.3% of instances correctly. This model may be clinically instructive in the medical monitoring and interventions in advance.

Most patients with COPD have other chronic diseases. For example, in a multicenter study in Poland, only 2% of patients with COPD have no comorbidities. Patients with COPD are most often associated with cardiovascular disease, diabetes, cerebrovascular disease, anxiety and depression^38^. On the one hand, patients with COPD are more likely to suffer from complications such as cardiovascular and cerebrovascular diseases due to smoking, systemic inflammatory response, hypoxemia, and decreased exercise tolerance. On the other hand, comorbidities can also affect the treatment and prognosis of patients^39^. Therefore, inflammation indicators and multiple comorbidities affect each other and are associated with the development of COPD patients. It is necessary to analyze these factors together.

This study included neutrophil counts, C-reactive protein, procalcitonin, and indicators closely related to systemic inflammation. In order to further explore the possible indicators of systemic inflammatory response in patients with COPD, the study also added eosinophils and lymphocyte counts, erythrocyte sedimentation rate. The results show that the vital signs, medical history data, comorbidity and inflammatory indicators of patients with COPD have a good predictive value for the prognosis of patients. All indicators in the study are simple and objective, which are suitable for clinical work.

**Figure 3:**
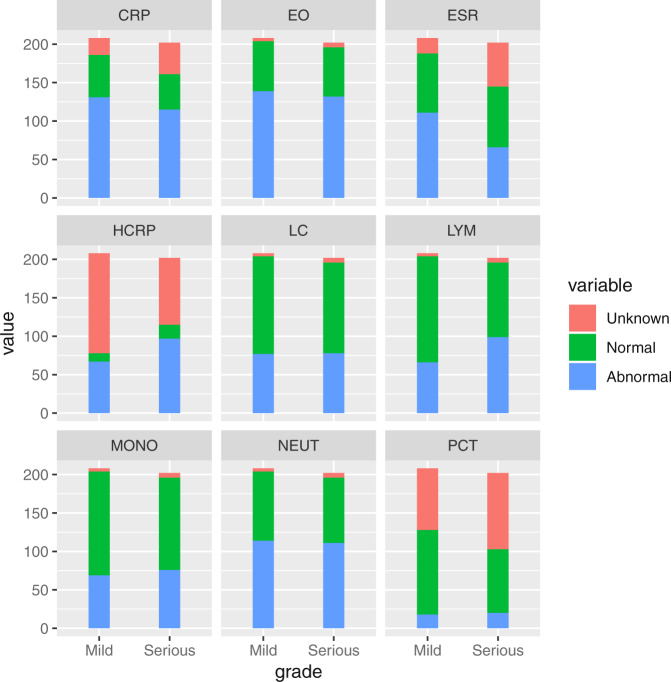
The result of medical laboratory of serious and mild COPD Patients. Note that CRP, EO, ESR, HCRP,LC, LYM, MONO, NEUT and PCT are C-reactive protein, absolute value of eosinophils, erythrocyte sedimentation rate, high sensitivity c-reactive protein, leukocyte count, lymphocyte absolute value, nuclear cell absolute value, absolute neutrophils and detection of procalcitonin. Unknown, Normal and Abnormal represents no test results, the normal results and abnormal results, respectively.

The 28 features we selected are simple, fast and objective. Measurements only require watches, sphygmomanometers, thermometers and routine clinical test. In clinical work, usually the nurses measure the 9 basic indicators (Table 4), ask for general information and register after admission, which takes 10 to 15 minutes. If we only specifically acquire the 9 indicators and use an electronic sphygmomanometer and an infrared thermometer, the time can be shortened to less than 5 minutes. 10 indicators describe the comorbidities of the COPD patients (Table 4) can be evaluated by doctors through the patients’ current and past medical history, which takes 20 to 30 minutes. 9 inflammation indicators (Fig. 3) from common laboratory testing, which takes 30 to 60 minutes. Total time spent on risk assessment is less than 2 hours. All clinical staff can quickly grasp this assessment method to assess the risk of deterioration for AECOPD. This assessment method can also be applied to outpatients, even patients self-assessment because of its easy operation.

**Table 4:** Distribution of mild and severe groups in patients with AECOPD Values are expressed as mean ± standard deviation.

		Mild Group(low risk)	Severe Group(high risk)
Number of cases		208 (50.7%)	202 (49.3%)
Sex	Male	163 (78.4%)	178 (88.1%)
	Female	39 (18.8%)	30 (14.9%)
Smoking history (SMK)		136 (65.4%)	163 (80.7%)
Age(year)		79 ± 9	82 ± 9
Number of hospitalizations (NOH)		3.8 ± 2.8	6.3 ± 7.3
Temperature (TEMP)		36.8 ± 0.5	36.7 ± 0.6
Pulse rate (PULSE)		92.5 ± 15.6	98.1 ± 17.6
Respiratory rate (RES)		21.6 ± 3.1	24.5 ± 6
Systolic pressure (SP)		133.2 ± 19.3	132.1 ± 24.7
Diastolic pressure (DP)		76.1 ± 12.2	74.5 ± 13.9
Pulmonary heart disease (PHD)	With	36 (17.3%)	65 (32.2%)
	Without	172 (82.7%)	137 (67.8%)
Bronchiectasis (BRCH)	With	14 (7%)	8 (4%)
	Without	194(93%)	194 (96%)
Hypertension (HTN)	With	86 (41.3%)	80 (39.6%)
	Without	122 (58.7%)	122 (60.4%)
Diabetes mellitus (DM)	With	17 (8.2%)	41 (20.3%)
	Without	191(91.8%)	161 (79.7%)
Coronary heart disease (CHD)	With	21 (10.1%)	36 (17.8%)
	Without	187 (89.9%)	166 (82.2%)
Chronic kidney disease (CKD)	With	4 (2%)	36 (2%)
	Without	204 (98%)	166 (98%)
Malignant tumor (MT)	With	16 (8%)	20 (10%)
	Without	192 (92%)	182 (90%)
cerebrovascular disease (CD)	With	9(4%)	8 (4%)
	Without	199 (96%)	194 (96%)
Viral hepatitis (HBV)	With	3 (1%)	4 (2%)
	Without	205 (99%)	198 (98%)
Cirrhosis (CRHS)	With	1 (0.5%)	1 (0.5%)
	Without	207 (99.5%)	201 (99.5%)

There are also deficiencies in this study, such as the lack of inclusion of serum cystatin, interleukin and other indicators of inflammation, because only a few patients have performed these tests. In addition, since this study is a retrospective analysis, there is no routine access to the patient’s BMI, as well as information on the anxiety and depression scale. Therefore, it is difficult to count comorbidities such as metabolic abnormalities, anxiety and depression. We will obtain a more complete medical history and indicators in further prospective studies. In addition, future research will be conducted by incorporating new features and comparing the model with the predictive performance of respiratory physicians.

## Methods

### Study population

This study was approved by the Third Affiliated Hospital, Sun Yat-sen University Institutional Review Board (IRB) with protocol #[2019]-02-334-01 and all enrolled individuals have provided written informed consent for collection, storage, make availability of their data for health-related research. All methods were carried out in accordance with the relevant guidelines and regulations. We removed the sensitive information of all patients before analysis, such as name, address, contact details, etc.

From 2011 to 2018, we select the patients with a history of COPD as the initial study population from the respiratory unit database of the Third Affiliated Hospital, Sun Yat-sen University (TAHSYU). More than 4,900 patients are enrolled in the preliminary study population. Since the purpose of our study is to achieve an early risk assessment for hospitalized AECOPD patients, we further identify 1954 discharged patients diagnosed with AECOPD from the preliminary study population as the intermediate study population. From the intermediate study population, we use the event of admission to the ICU to distinguish severe and mild patients. The intermediate study population consisted of 244 AECOPD patients those required the admission to the ICU and 1710 AECOPD patients those who had not been in the ICU. Among the 244 patients, 42 AECOPD patients were excluded for lack of key indicators. Then, 202 severe AECOPD patients those required the admission to the ICU were labeled as severe patients. 208 AECOPD patients extracted from 1710 AECOPD patients using undersampling technology were labeled as mild patients.

Finally, we identify 410 hospital records from the adult patients (age > 18 years) with AECOPD by using International Classification of Diseases, Tenth Revision,Clinical Modification (ICD-10-CM) code for COPD(J44.100, J44.101) in the primary diagnosis field^40,41^. For statistical convenience, we define patients those require the admission to the ICU as severe, while those don’t require as mild. The proportion of patients with serious AECOPD is 49.3%, while the proportion of mild is 50.7%.

To analyze the impact of base indicators, comorbidities, and inflammation on the frequent severe acute exacerbations (AEs) in patients with COPD, we implement the following approach: the data extracted from the the respiratory unit database of TAHSYU are randomly split into a training set (328 people) and a testing set (82 people). Samples with too many missing values are excluded to ensure an unbiased risk assessment for acute exacerbations (AEs) in patients with COPD. Distribution of mild and severe groups in patients with AECOPD values is shown in Table 4. As indicated in Table 4, out of 410 patients, 208 of them with COPD are successfully diagnosed stable COPD, while 202 patients are diagnosed as acute exacerbation of COPD. 299 patients has a history of smoking (136 patients belong to the serious group while 163 patients belong to the mild group), while 111 patients don’t have smoking history (66 patients belong to the serious group while 45 patients belong to the mild group). 341 patients are male (163 patients belong to the serious group while 178 patients belong to the mild group) and 69 patients are female (39 patients belong to the serious group while 30 patients belong to the mild group). Out of 410 patients, 283 patients (69%) have at least one comorbidity, 127 patients (31%) have no comorbidities.

### Data feature selection

In this paper, the process of feature selection is culled in the following multiple steps: (1) to ensure the validity and rationality from the clinical perspective; (2) to verify the relatively comprehensive from the perspective of system engineering; (3) to guarantee the selected features are structured. The unstructured features like past history, current history, course of disease are left for follow-up study.

Finally, we select 28 features that are most clinically relevant. Among the selected features, 9 features (sex, age, smoking history, number of hospitalization, temperature, pulse rate, respiratory rate, systolic blood pressure, and diastolic blood pressure) are the most basic characteristics of the COPD patients (Table 4). 10 features describe the comorbidities of the COPD patients (Table 4). According to the correlation with COPD, we label pulmonary heart disease and bronchiectasis as the most critical factors, followed by hypertension, diabetes and coronary heart disease, and the remaining markers are the most common factors. 9 features indicate the inflammation of the COPD patients (Fig. 3).

### Classifiction using C5.0 model

At this stage, 28 features which are extracted from 410 COPD patients’ records are shown in Table 1 and Fig. 2. From the available data set, each of the *N* observations is denoted by the 2-tuple, (*x*, *y*), where *x* ∈ {*x*_1_, *x*_2_, …, *x*_28_} is the vector containing all the selected features. *y* ∈ {1, 2} represents the categories of ‘low risk’ and ‘high risk’.

To analyze the impact of the actual machine-learning approach in a further investigation, we use 80% of the observation for model training, while the remaining 20% for model validation. Entropy is a measure of the uncertainty of a random variable. In a binary classification system, the entropy of the system can be expressed as:

5 $$H(y)=-{\sum }_{i=1}^{2}{p}_{(y=i)}{{\rm{\log }}}_{2}{p}_{(y=i)},$$

where p(y = 1) and p(y = 2) are respectively the probability of low risk and high risk of the AECOPD patients. Similarly, the entropy of feature *x* can be expressed as:

6 $$H(x)=-{\sum }_{i=1}^{n}{p}_{i}{{\rm{\log }}}_{2}{p}_{i},$$

where *p*_*i*_ = *P*(*X* = *x*_*i*_) is the probability distribution of feature *x*. Information entropy is a most commonly used index to measure the purity of a sample set. The feature with large information gain is more likely to be selected as a node which tend to be closer to the root node.

The information gain is the reduction of uncertainty of *Y* under certain feature *X*. In addition, the infomation gain of feature *x* can be computed as:

7 $$IG(y,x)=H(y)-H(y| x),$$

where *H*(*y*) is the entropy of feature *y*, *H*(*y*∣*x*) is the conditional entropy which is used to measure the uncertainty of a variable y given a certain value of x, $H(y| x)=-{\sum }_{i=1}^{n}p(y,{x}_{i}){{\rm{\log }}}_{2}p(y| x={x}_{i})$. The information gain *I* *G*(*y*, *x*) is used to evaluate the optimal branching features and segmentation threshold, and the decrease of information entropy means the decrease of uncertainty. However, information gain tends to select features with more values, which is likely to lead to overfitting.

Information gain ratio is proposed to overcome the deciency of information gain. The information gain rate is expressed as:

8 $$IGR(y,x)=\frac{IG(y,x)}{H(y| x)}.$$

All features are evaluated by information gain ratio *I* *G* *R*(*y*, *x*) to find the optimal branching features with the maximum *I* *G* *R*(*y*, *x*) values. The messier the category overall, the bigger the *I* *G* *R*(*y*, *x*) value (or vice versa). The splitting process is repeated until all leaf nodes reside no greater than the predefined depth from the root node for all existing leaf nodes. A cross-validated gridsearch approach is applied to tune the hyperparameters.

A boosting procedure is employed to improve the prediction performance by constructing multiple decision tree models. Early stopping approach is used to enhance the generalization ability for each decision tree. The first weak C5.0 classifiers is created. This method is similar to adaptive boost algorithm. When the first weak decision tree is created, it will make the prediction for the sample. If one sample is misclassified, its weight will be increased, and the next weak decision tree pays more attention to this sample; otherwise, its weight will be decreased. Finally, the final prediction results are generated by combining the outputs of the multiple weak decision tesss using a weighted majority vote. Further, early stopping approach is used to enhance the generalization ability for each decision tree.

Specially, In the process of node splitting, both C5.0 and C4.5 adopt information gain ratio, while CART adopts gini index and (iterative dichotomiser 3) ID3 adopts information gain. The ID3 algorithm cannot handle attributes with continuous values and with missing values. For attributes with many values, the ID3 algorithm is very sensitive. The C4.5 algorithm solves this problem by using the gain rate. Classification and Regression Trees (CART) are very similar to the C4.5 algorithm, while CART supports predicting continuous values (Regression). CART generally builds only binary trees, while C4.5 can build other trees. Compared with C4.5 algorithm, C5.0 supports boosting, uses less memory, builds smaller rule sets, runs faster and has stronger generality ability. Under the premise of obtaining the same prediction accuracy, the decision tree established by C5.0 is generally smaller than that by C4.5. We implement C5.0, CART and ID3 classifiers on the platform of R3.5.1.

## Supplementary information

Supplementary Information.

## Data Availability

The data sets generated during and/or analyzed during the current study are available from the corresponding author on reasonable request.
