# Author Correction: A Machine-learning Approach to Forecast Aggravation Risk in Patients with Acute Exacerbation of Chronic Obstructive Pulmonary Disease with Clinical Indicators

**DOI:** 10.1038/s41598-020-76434-2

**Published:** 2021-03-02

**Authors:** Junfeng Peng, Chuan Chen, Mi Zhou, Xiaohua Xie, Yuqi Zhou, Ching-Hsing Luo

**Affiliations:** 1 School of Data and Computer Science, Sun Yat-sen University, Guangzhou, 510006 China; 2 The Third Affiliated Hospital, Sun Yat-sen University, Guangzhou, 510640 China

Correction to: *Scientific Reports,* 10.1038/s41598-020-60042-1, Published on 20 February 2020

The Article contains errors in Table 4 where the reported results for the “Mild Group (low risk)” and “Severe Group (high risk)” are incorrect.

The results for the Mild Group (low risk) should read Male 178 (85.6%), Female 30 (14.4%) and Smoking history (SMK) 163 (78.4%).

The results for the Severe Group (high risk) should read Male 163 (80.7%), Female 39 (19.3%) and Smoking history (SMK) 136 (67.3%).

The correct Table 4 appears below as Table 1.

**Table 1:** A correct version of the original Table 4.

		Mild group (low risk)	Severe group (high risk)
Number of cases		208 (50.7%)	202 (49.3%)
Sex	Male	178 (85.6%))	163 (80.7%)
	Female	30 (14.4%)	39 (19.3%)
Smoking history (SMK)		163 (78.4%)	136 (67.3%)
Age (year)		79 ± 9	82 ± 9
Number of hospitalizations (NOH)		3.8 ± 2.8	6.3 ± 7.3
Temperature (TEMP)		36.8 ± 0.5	36.7 ± 0.6
Pulse rate (PULSE)		92.5 ± 15.6	98.1 ± 17.6
Respiratory rate (RES)		21.6 ± 3.1	24.5 ± 6
Systolic pressure (SP)		133.2 ± 19.3	132.1 ± 24.7
Diastolic pressure (DP)		76.1 ± 12.2	74.5 ± 13.9
Pulmonary heart disease (PHD)	With	36 (17.3%)	65 (32.2%)
	Without	172 (82.7%)	137 (67.8%)
Bronchiectasis (BRCH)	With	14 (7%)	8 (4%)
	Without	194 (93%)	194 (96%)
Hypertension (HTN)	With	86 (41.3%)	80 (39.6%)
	Without	122 (58.7%)	122 (60.4%)
Diabetes mellitus (DM)	With	17 (8.2%)	41 (20.3%)
	Without	191 (91.8%)	161 (79.7%)
Coronary heart disease (CHD)	With	21 (10.1%)	36 (17.8%)
	Without	187 (89.9%)	166 (82.2%)
Chronic kidney disease (CKD)	With	4 (2%)	36 (2%)
	Without	204 (98%)	166 (98%)
Malignant tumor (MT)	With	16 (8%)	20 (10%)
	Without	192 (92%)	182 (90%)
Cerebrovascular disease (CD)	With	9 (4%)	8 (4%)
	Without	199 (96%)	194 (96%)
Viral hepatitis (HBV)	With	3 (1%)	4 (2%)
	Without	205 (99%)	198 (98%)
Cirrhosis (CRHS)	With	1 (0.5%)	1 (0.5%)
	Without	207 (99.5%)	201 (99.5%)

