# Supplementary material for: A Machine-learning Approach to Forecast Aggravation Risk in Patients with Acute Exacerbation of Chronic Obstructive Pulmonary Disease with Clinical Indicators
Source: Sci Rep. 2020 Feb 20;10:3118. doi: 10.1038/s41598-020-60042-1 (PMC7033165; doi:10.1038/s41598-020-60042-1)
Supplement: Supplementary file 1 — Supplementary Information. [file 41598_2020_60042_MOESM1_ESM.docx]

A Machine-learning Approach to Forecast Aggravation Risk in Patients with Acute Exacerbation of Chronic Obstructive Pulmonary Disease with Clinical Indicators

JunfengPeng^a^ Msc, Chuan Chen^a^ Ph.D., Mi Zhou^c^ M.D., XiaohuaXie^*a^ Ph.D.,
YuqiZhou^∗b^ M.D.,Ph.D, Ching-HsingLuo^a^ M.D.

*^a^School of Data and Computer Science, Sun Yat-sen University, Guangzhou 510006,China*

*^b^Department of respiratory and critical care medicine, The Third Affiliated Hospital of Sun Yat-sen University, Guangzhou 510640, China*

**The summary of the C5.0 classifier (40 decision trees):**

summary(plsFit_c50)

Call:

(function (x, y, trials = 1, rules = FALSE, weights = NULL, control

FALSE, noGlobalPruning = FALSE, CF = 0.25, minCases = 2, fuzzyThreshold

= FALSE, sample = 0, earlyStopping = TRUE, label = "outcome", seed = 980L))

C5.0 [Release 2.07 GPL Edition] Tue Dec 24 22:13:44 2019

-------------------------------

Class specified by attribute `outcome'

Read 329 cases (38 attributes) from undefined.data

----- Trial 0: -----

Decision tree:

NOH > 1.78983: 2 (21/1)

NOH <= 1.78983:

:...RES > 0.7058755:

:...LYM1 <= -1.111005: 2 (23/1)

: LYM1 > -1.111005:

: :...EO1 <= -0.6598135:

: :...AGE <= 0.6519183: 1 (5)

: : AGE > 0.6519183: 2 (3)

: EO1 > -0.6598135:

: :...NEUT1 <= -0.854023: 2 (9)

: NEUT1 > -0.854023:

: :...CRP2 <= -1.275555: 2 (6/1)

: CRP2 > -1.275555: 1 (2)

RES <= 0.7058755:

:...PCT1 <= -0.9715286:

:...ESR2 > -0.8700863:

: :...EO1 <= -0.6598135: 1 (39/6)

: : EO1 > -0.6598135:

: : :...CRP2 <= -1.275555: 1 (4)

: : CRP2 > -1.275555:

: : :...MONO1 <= -1.292171: 1 (4/1)

: : MONO1 > -1.292171: 2 (11)

: ESR2 <= -0.8700863:

: :...DM2 > -0.3974532: 2 (9)

: DM2 <= -0.3974532:

: :...HCRP2 > -0.8173213:

: :...NEUT1 <= -0.854023: 2 (11)

: : NEUT1 > -0.854023:

: : :...DP <= -0.09096502: 2 (2)

: : DP > -0.09096502: 1 (2)

: HCRP2 <= -0.8173213:

: :...CD2 > -0.3974532: 1 (4)

: CD2 <= -0.3974532:

: :...TEMP > 1.21706: 2 (7)

: TEMP <= 1.21706:

: :...TEMP <= -0.8145545:

: :...HTN2 <= -0.8381655: 2 (9)

: : HTN2 > -0.8381655:

: : :...AGE <= 0.1191783: 1 (2)

: : AGE > 0.1191783: 2 (2)

: TEMP > -0.8145545:

: :...MONO1 <= -1.292171:

: :...PHD3 > -0.556608: 2 (3)

: : PHD3 <= -0.556608:

: : :...SP <= 0.1398637: 1 (6)

: : SP > 0.1398637: 2 (7/1)

: MONO1 > -1.292171:

: :...HTN2 <= -0.8381655: 1 (12/1)

: HTN2 > -0.8381655:

: :...RES <= -0.8060583: 1 (2)

: RES > -0.8060583: 2 (3)

PCT1 > -0.9715286:

:...LYM1 > -1.111005:

:...PHD3 <= -0.556608: 1 (67/6)

: PHD3 > -0.556608:

: :...HCRP1 > -0.2800881: 2 (2)

: HCRP1 <= -0.2800881:

: :...AGE <= 0.7584663: 1 (4)

: AGE > 0.7584663: 2 (4/1)

LYM1 <= -1.111005:

:...EO1 > -0.6598135: 1 (8/1)

EO1 <= -0.6598135:

:...SMK <= -1.67839: 2 (10/2)

SMK > -1.67839:

:...BRCH3 > -0.2330699: 2 (5/1)

BRCH3 <= -0.2330699:

:...PHD3 > -0.556608: 1 (5)

PHD3 <= -0.556608:

:...MONO1 <= -1.292171: 2 (3)

MONO1 > -1.292171:

:...PULSE > 1.187347: 2 (3)

PULSE <= 1.187347:

:...CD2 <= -0.3974532: 1 (6)

CD2 > -0.3974532:

:...SP <= 0.8896013: 2 (2)

SP > 0.8896013: 1 (2)

----- Trial 1: -----

Decision tree:

HCRP1 > -0.2800881: 2 (28.4/6.9)

HCRP1 <= -0.2800881:

:...DP > 0.3815743:

:...MONO1 <= -1.292171:

: :...AGE <= -1.372494: 1 (6.4)

: : AGE > -1.372494:

: : :...CVD1 > -0.2025196: 1 (2.3)

: : CVD1 <= -0.2025196:

: : :...WBC2 <= -0.7917082: 2 (15.6/3.1)

: : WBC2 > -0.7917082: 1 (11.5/3.8)

: MONO1 > -1.292171:

: :...PULSE <= -0.04893969: 1 (28.9/3.8)

: PULSE > -0.04893969:

: :...CRP1 > -0.556608: 2 (3.8)

: CRP1 <= -0.556608:

: :...AGE <= 0.9715623: 1 (27.6/5.4)

: AGE > 0.9715623: 2 (3.1)

DP <= 0.3815743:

:...NOH > 1.78983: 2 (12.3)

NOH <= 1.78983:

:...PCT1 > -0.9715286:

:...SP > 1.170753: 2 (9.7)

: SP <= 1.170753:

: :...RES <= -0.1580867: 1 (42/3.8)

: RES > -0.1580867:

: :...CD2 > -0.3974532: 2 (3.1)

: CD2 <= -0.3974532:

: :...HCRP2 <= -0.8173213: 1 (6.2/1.5)

: HCRP2 > -0.8173213: 2 (16.6/5.4)

PCT1 <= -0.9715286:

:...PCT2 > -0.3162744:

:...TEMP <= 0.4782911: 1 (12.3/3.8)

: TEMP > 0.4782911: 2 (8.7/0.8)

PCT2 <= -0.3162744:

:...DM2 > -0.3974532: 2 (5.4)

DM2 <= -0.3974532:

:...LYM2 > -0.8487149: 2 (43/8.5)

LYM2 <= -0.8487149:

:...NOH > -0.1813796: 2 (12)

NOH <= -0.1813796:

:...HTN2 > -0.8381655: 1 (6.2)

HTN2 <= -0.8381655:

:...ESR2 <= -0.8700863: 2 (16.4/3.1)

ESR2 > -0.8700863: 1 (7.7/1.5)

----- Trial 2: -----

Decision tree:

RES > 1.137856:

:...PULSE <= -1.167485: 1 (3.9/0.6)

: PULSE > -1.167485: 2 (26.6/1.8)

RES <= 1.137856:

:...DM2 > -0.3974532:

:...HCRP1 > -0.2800881: 2 (8.3)

: HCRP1 <= -0.2800881:

: :...HCRP2 <= -0.8173213: 1 (8.6/2.4)

: HCRP2 > -0.8173213:

: :...TEMP <= -0.6298622: 1 (6.7/1.2)

: TEMP > -0.6298622: 2 (19.7/1.2)

DM2 <= -0.3974532:

:...HCRP2 > -0.8173213:

:...DP > 1.247896: 1 (6.3)

: DP <= 1.247896:

: :...TEMP > 1.21706: 1 (7.3/0.6)

: TEMP <= 1.21706:

: :...ESR2 <= -0.8700863: 2 (40.6/6.7)

: ESR2 > -0.8700863:

: :...CRP1 > -0.556608: 2 (6.7/1.2)

: CRP1 <= -0.556608:

: :...MT1 > -0.3220414: 2 (3.9/0.6)

: MT1 <= -0.3220414:

: :...TEMP > 0.4782911: 2 (7.1/0.6)

: TEMP <= 0.4782911:

: :...NOH <= 1.610629: 1 (19.7/1.8)

: NOH > 1.610629: 2 (2.4)

HCRP2 <= -0.8173213:

:...CD2 > -0.3974532: 1 (8.7/0.6)

CD2 <= -0.3974532:

:...TEMP > 0.8476756:

:...PCT1 <= -0.9715286: 2 (14.6/1.8)

: PCT1 > -0.9715286: 1 (2.4)

TEMP <= 0.8476756:

:...TEMP <= -0.8145545:

:...NOH <= -0.7189821: 2 (7.9)

: NOH > -0.7189821:

: :...PHD3 > -0.556608: 2 (5.5/0.6)

: PHD3 <= -0.556608:

: :...TEMP <= -1.183939: 1 (9.1/0.6)

: TEMP > -1.183939: 2 (4.9/1.2)

TEMP > -0.8145545:

:...TEMP > 0.6629834: 1 (12.2)

TEMP <= 0.6629834:

:...NEUT1 > -0.854023: 1 (29.2/3.7)

NEUT1 <= -0.854023:

:...NEUT2 <= -1.104199: 2 (5.9/1.8)

NEUT2 > -1.104199:

:...PULSE <= -1.108614: 2 (3.2)

PULSE > -1.108614:

:...HCRP1 > -0.2800881: 1 (6.4)

HCRP1 <= -0.2800881:

:...AGE <= -0.9463016: 2 (9.1/1.2)

AGE > -0.9463016: 1 (42/10.5)

----- Trial 3: -----

Decision tree:

DM2 > -0.3974532:

:...PCT2 > -0.3162744: 1 (4.6/1.4)

: PCT2 <= -0.3162744:

: :...PCT1 <= -0.9715286: 2 (12.6)

: PCT1 > -0.9715286:

: :...TEMP <= -0.8145545: 1 (4.3)

: TEMP > -0.8145545: 2 (25.6/5)

DM2 <= -0.3974532:

:...AGE > 0.9715623:

:...SEX <= -2.204582: 1 (9.6/3.6)

: SEX > -2.204582: 2 (31.6/4.5)

AGE <= 0.9715623:

:...LYM2 <= -0.8487149:

:...AGE <= -1.372494: 1 (19.4)

: AGE > -1.372494:

: :...PHD3 > -0.556608:

: :...MONO1 <= -1.292171: 1 (3.1/0.5)

: : MONO1 > -1.292171: 2 (10.4/2.6)

: PHD3 <= -0.556608:

: :...ESR2 > -0.8700863:

: :...SP <= 0.7958841: 1 (29.9/0.5)

: : SP > 0.7958841: 2 (7.4/1.9)

: ESR2 <= -0.8700863:

: :...NEUT1 > -0.854023:

: :...NOH <= 0.5354238: 1 (23.7/2.4)

: : NOH > 0.5354238: 2 (2.6)

: NEUT1 <= -0.854023:

: :...EO1 <= -0.6598135: 2 (19/3.8)

: EO1 > -0.6598135:

: :...RES <= 0.9218659: 1 (9)

: RES > 0.9218659: 2 (3.1)

LYM2 > -0.8487149:

:...RES > 1.137856: 2 (9.4)

RES <= 1.137856:

:...MT1 > -0.3220414: 2 (6.5/1)

MT1 <= -0.3220414:

:...CRP2 <= -1.275555:

:...BRCH3 > -0.2330699: 2 (3)

: BRCH3 <= -0.2330699:

: :...RES <= 0.4898849: 1 (20.1/2.9)

: RES > 0.4898849: 2 (2.2)

CRP2 > -1.275555:

:...WBC1 <= -1.189455:

:...EO1 > -0.6598135: 2 (9.3/3)

: EO1 <= -0.6598135:

: :...DP <= -0.799774: 2 (5.9/1.3)

: DP > -0.799774:

: :...TEMP <= 0.8476756: 1 (13.9/0.5)

: TEMP > 0.8476756: 2 (2.6)

WBC1 > -1.189455:

:...DP > 1.247896: 1 (4)

DP <= 1.247896:

:...CVD1 > -0.2025196: 2 (2.7)

CVD1 <= -0.2025196:

:...HCRP2 > -0.8173213: 2 (15.2/1.4)

HCRP2 <= -0.8173213:

:...PCT1 <= -0.9715286: 2 (12.7/2.2)

PCT1 > -0.9715286: 1 (5.6)

----- Trial 4: -----

Decision tree:

RES > 0.4898849: 2 (60/17.7)

RES <= 0.4898849:

:...NOH > 1.252227:

:...EO1 <= -0.6598135: 2 (15/1.6)

: EO1 > -0.6598135: 1 (3)

NOH <= 1.252227:

:...PCT1 > -0.9715286:

:...LYM1 <= -1.111005:

: :...SP > 0.7021669: 2 (16.5/2.3)

: : SP <= 0.7021669:

: : :...AGE <= -1.58559: 2 (3.3)

: : AGE > -1.58559:

: : :...CD2 <= -0.3974532: 1 (19.6/1.4)

: : CD2 > -0.3974532: 2 (4.9/1.6)

: LYM1 > -1.111005:

: :...DP > -0.3272347: 1 (41.3/0.8)

: DP <= -0.3272347:

: :...SP <= -0.6567326: 1 (12.4)

: SP > -0.6567326:

: :...CRP2 <= -1.275555: 2 (11.3/1)

: CRP2 > -1.275555: 1 (11.2/4)

PCT1 <= -0.9715286:

:...DM2 > -0.3974532: 2 (13/2.1)

DM2 <= -0.3974532:

:...SEX <= -2.204582:

:...AGE <= 1.291206: 2 (10.7/1.7)

: AGE > 1.291206: 1 (2.7)

SEX > -2.204582:

:...MT1 > -0.3220414:

:...PCT2 > -0.3162744: 1 (2.9)

: PCT2 <= -0.3162744:

: :...AGE <= 0.4388223: 1 (4.9/1)

: AGE > 0.4388223: 2 (11.8)

MT1 <= -0.3220414:

:...BRCH3 > -0.2330699: 1 (4.6)

BRCH3 <= -0.2330699:

:...HTN2 > -0.8381655: 1 (18.7/3.8)

HTN2 <= -0.8381655:

:...TEMP <= -0.8145545: 2 (6.2/0.4)

TEMP > -0.8145545:

:...CRP2 <= -1.275555: 1 (12.9/1.9)

CRP2 > -1.275555:

:...SP > -0.04757074: 2 (10.7/0.4)

SP <= -0.04757074:

:...PULSE <= 0.2454142: 1 (22.6/3.4)

PULSE > 0.2454142: 2 (8.8/2.7)

----- Trial 5: -----

Decision tree:

SMK <= -1.67839:

:...CKD1 > -0.1472177: 1 (4.6)

: CKD1 <= -0.1472177:

: :...MONO1 <= -1.292171: 2 (24.3/4.3)

: MONO1 > -1.292171:

: :...PCT1 <= -0.9715286:

: :...MT1 <= -0.3220414: 2 (19.7/3.7)

: : MT1 > -0.3220414: 1 (2)

: PCT1 > -0.9715286:

: :...SP > 0.8427427: 2 (8.9/1)

: SP <= 0.8427427:

: :...HBV1 <= -0.1472177: 1 (24.3/2.7)

: HBV1 > -0.1472177: 2 (2.8/0.3)

SMK > -1.67839:

:...MT1 > -0.3220414:

:...NOH <= -0.3605804: 1 (10.3/2.9)

: NOH > -0.3605804: 2 (12/0.6)

MT1 <= -0.3220414:

:...RES > 1.353847: 2 (19.5/6.2)

RES <= 1.353847:

:...HTN2 > -0.8381655:

:...LYM2 <= -0.8487149:

: :...CKD1 <= -0.1472177: 1 (43.6/2.5)

: : CKD1 > -0.1472177: 2 (2.6)

: LYM2 > -0.8487149:

: :...BRCH3 > -0.2330699: 2 (3.1)

: BRCH3 <= -0.2330699:

: :...PCT1 <= -0.9715286: 2 (6.3/0.8)

: PCT1 > -0.9715286: 1 (18/3.7)

HTN2 <= -0.8381655:

:...AGE <= -0.8397537: 1 (37.3/8.1)

AGE > -0.8397537:

:...NOH > -0.1813796: 2 (22.4/2.3)

NOH <= -0.1813796:

:...PCT1 > -0.9715286: 1 (13.3/0.8)

PCT1 <= -0.9715286:

:...EO1 > -0.6598135:

:...SP <= -0.375581: 2 (8.3)

: SP > -0.375581: 1 (6/1.7)

EO1 <= -0.6598135:

:...CRP1 > -0.556608: 2 (5.9/1.2)

CRP1 <= -0.556608:

:...CRP2 <= -1.275555: 1 (2.1)

CRP2 > -1.275555:

:...TEMP > 0.4782911: 2 (5.1/1)

TEMP <= 0.4782911:

:...ESR2 > -0.8700863: 1 (11)

ESR2 <= -0.8700863:

:...MONO1 <= -1.292171: 2 (2.7)

MONO1 > -1.292171: 1 (12.7/3.5)

----- Trial 6: -----

Decision tree:

PULSE > 0.8341221:

:...SEX <= -2.204582: 2 (6.4)

: SEX > -2.204582:

: :...SMK <= -1.67839: 1 (4.3/0.5)

: SMK > -1.67839:

: :...MONO1 > -1.292171: 2 (31.5/3.3)

: MONO1 <= -1.292171:

: :...PULSE > 1.717184: 2 (3.7)

: PULSE <= 1.717184:

: :...RES <= 0.7058755: 1 (12.1/0.9)

: RES > 0.7058755: 2 (4.5/1.1)

PULSE <= 0.8341221:

:...NOH > 0.8938254: 2 (14.9/2.2)

NOH <= 0.8938254:

:...LYM1 > -1.111005:

:...DP > 0.5390874: 1 (41.9/3.4)

: DP <= 0.5390874:

: :...SMK <= -1.67839:

: :...HTN2 > -0.8381655: 2 (14/2.1)

: : HTN2 <= -0.8381655:

: : :...DP <= -0.5635043: 2 (8/0.9)

: : DP > -0.5635043: 1 (9)

: SMK > -1.67839:

: :...HCRP1 > -0.2800881: 1 (5.4)

: HCRP1 <= -0.2800881:

: :...NOH > -0.002178733: 2 (10.1/1.7)

: NOH <= -0.002178733:

: :...MONO1 <= -1.292171: 1 (13.9)

: MONO1 > -1.292171:

: :...CKD1 > -0.1472177: 2 (2.1)

: CKD1 <= -0.1472177:

: :...PCT1 > -0.9715286: 1 (12.7)

: PCT1 <= -0.9715286:

: :...ESR2 <= -0.8700863: 2 (6/0.9)

: ESR2 > -0.8700863: 1 (13.9/5.3)

LYM1 <= -1.111005:

:...RES > 1.137856: 2 (6)

RES <= 1.137856:

:...ESR2 <= -0.8700863:

:...LYM2 <= -0.8487149: 2 (3.5)

: LYM2 > -0.8487149:

: :...BRCH3 > -0.2330699: 2 (3)

: BRCH3 <= -0.2330699:

: :...HCRP2 > -0.8173213: 2 (13/1.6)

: HCRP2 <= -0.8173213:

: :...PCT1 > -0.9715286: 1 (5.3)

: PCT1 <= -0.9715286:

: :...SP <= -0.8910256: 1 (6.7)

: SP > -0.8910256:

: :...SP <= -0.4224396: 2 (11.3)

: SP > -0.4224396:

: :...TEMP <= -0.8145545: 2 (3.9)

: TEMP > -0.8145545: 1 (15/4.4)

ESR2 > -0.8700863:

:...PCT2 > -0.3162744: 1 (8.4)

PCT2 <= -0.3162744:

:...SEX <= -2.204582: 2 (3.6/0.2)

SEX > -2.204582:

:...DM2 > -0.3974532: 1 (3.9)

DM2 <= -0.3974532:

:...LYM2 <= -0.8487149: 1 (3.8)

LYM2 > -0.8487149:

:...BRCH3 > -0.2330699: 1 (2.2)

BRCH3 <= -0.2330699:

:...EO1 > -0.6598135: 1 (6.2/0.8)

EO1 <= -0.6598135:

:...SMK <= -1.67839: 1 (5.7/1.4)

SMK > -1.67839:

:...RES <= 0.2738944: 2 (10.3/1.5)

RES > 0.2738944: 1 (2.9)

----- Trial 7: -----

Decision tree:

PCT1 > -0.9715286:

:...HCRP2 <= -0.8173213:

: :...HCRP1 <= -0.2800881:

: : :...AGE <= 0.9715623: 1 (48.2/4.2)

: : : AGE > 0.9715623: 2 (2.6)

: : HCRP1 > -0.2800881:

: : :...MONO1 <= -1.292171: 2 (3.9)

: : MONO1 > -1.292171: 1 (10.5/4.2)

: HCRP2 > -0.8173213:

: :...CD2 > -0.3974532: 2 (17.2/1.6)

: CD2 <= -0.3974532:

: :...BRCH3 > -0.2330699: 2 (3.7/0.6)

: BRCH3 <= -0.2330699:

: :...SP > 1.26447: 2 (5.6)

: SP <= 1.26447:

: :...CRP2 > -1.275555: 1 (32.6/6.4)

: CRP2 <= -1.275555:

: :...DM2 > -0.3974532: 2 (6.8)

: DM2 <= -0.3974532:

: :...NOH <= 0.5354238: 1 (12.7/2.1)

: NOH > 0.5354238: 2 (2.1)

PCT1 <= -0.9715286:

:...ESR2 > -0.8700863:

:...EO1 <= -0.6598135: 1 (44.1/10.2)

: EO1 > -0.6598135:

: :...MONO1 <= -1.292171: 1 (7.5/2)

: MONO1 > -1.292171:

: :...RES <= 1.137856: 2 (21.4/0.8)

: RES > 1.137856: 1 (3.1/0.4)

ESR2 <= -0.8700863:

:...TEMP > 1.401752: 2 (7.3)

TEMP <= 1.401752:

:...EO1 > -0.6598135:

:...HCRP2 > -0.8173213: 2 (2.7)

: HCRP2 <= -0.8173213:

: :...SP > 0.6084497: 1 (9.4)

: SP <= 0.6084497:

: :...SP <= -0.609874: 1 (7.2/0.4)

: SP > -0.609874: 2 (14/3.1)

EO1 <= -0.6598135:

:...ESR1 <= -0.8070197: 2 (15.4/1.8)

ESR1 > -0.8070197:

:...NOH > -0.1813796: 2 (6.7)

NOH <= -0.1813796:

:...RES > 0.05790384: 1 (11/1.8)

RES <= 0.05790384:

:...SMK <= -1.67839: 2 (5.8)

SMK > -1.67839:

:...RES <= -1.022049: 1 (5.4)

RES > -1.022049: 2 (22/5)

----- Trial 8: -----

Decision tree:

NOH > 1.78983:

:...AGE <= -0.7332057: 1 (2.2)

: AGE > -0.7332057: 2 (14.5)

NOH <= 1.78983:

:...RES > 0.7058755:

:...LYM1 <= -1.111005: 2 (23.1/1.5)

: LYM1 > -1.111005:

: :...HTN2 > -0.8381655: 2 (12.4/1.5)

: HTN2 <= -0.8381655:

: :...NOH <= -0.7189821: 2 (4.1)

: NOH > -0.7189821: 1 (14/3.1)

RES <= 0.7058755:

:...MT1 > -0.3220414:

:...SMK <= -1.67839: 1 (6/0.8)

: SMK > -1.67839:

: :...MONO2 <= -0.7270632: 2 (15.6/2.3)

: MONO2 > -0.7270632: 1 (3.1/0.8)

MT1 <= -0.3220414:

:...NEUT1 > -0.854023:

:...PHD3 > -0.556608: 2 (13.8/4)

: PHD3 <= -0.556608:

: :...LYM1 > -1.111005:

: :...AGE <= 1.07811: 1 (36.9/4.6)

: : AGE > 1.07811: 2 (2.4)

: LYM1 <= -1.111005:

: :...TEMP <= -0.6298622: 2 (4)

: TEMP > -0.6298622:

: :...CRP2 <= -1.275555: 1 (6)

: CRP2 > -1.275555:

: :...RES <= -0.8060583: 2 (4.5)

: RES > -0.8060583:

: :...TEMP <= 0.1089067: 1 (10.3)

: TEMP > 0.1089067: 2 (8.1/1.7)

NEUT1 <= -0.854023:

:...ESR1 > -0.8070197:

:...NOH <= -0.7189821: 2 (6.4)

: NOH > -0.7189821:

: :...AGE <= -0.5201097:

: :...SP <= -1.125319: 1 (2.1)

: : SP > -1.125319: 2 (21.6/2.6)

: AGE > -0.5201097:

: :...WBC1 > -1.189455: 1 (7.7)

: WBC1 <= -1.189455:

: :...HCRP2 > -0.8173213: 2 (5.3/0.5)

: HCRP2 <= -0.8173213:

: :...MONO1 <= -1.292171: 2 (5.5/1.4)

: MONO1 > -1.292171: 1 (7.4)

ESR1 <= -0.8070197:

:...WBC1 > -1.189455:

:...CRP1 > -0.556608: 2 (3.6)

: CRP1 <= -0.556608:

: :...CRP2 <= -1.275555: 1 (4.1/0.1)

: CRP2 > -1.275555: 2 (20.7/6.6)

WBC1 <= -1.189455:

:...MONO2 > -0.7270632: 1 (23.7/2.7)

MONO2 <= -0.7270632:

:...BRCH3 > -0.2330699: 1 (2.1)

BRCH3 <= -0.2330699:

:...NOH > -0.1813796: 1 (6.9/0.6)

NOH <= -0.1813796:

:...ESR2 <= -0.8700863: 2 (4)

ESR2 > -0.8700863:

:...WBC2 <= -0.7917082: 1 (2.9)

WBC2 > -0.7917082:

:...TEMP <= -0.8145545: 1 (4.8)

TEMP > -0.8145545:

:...NOH <= -0.7189821: 1 (2.2)

NOH > -0.7189821: 2 (17/2.3)

----- Trial 9: -----

Decision tree:

RES > 0.7058755:

:...HBV1 > -0.1472177: 2 (4.5)

: HBV1 <= -0.1472177:

: :...RES > 2.217809: 2 (11.5)

: RES <= 2.217809:

: :...ESR1 <= -0.8070197: 2 (17.6/2.6)

: ESR1 > -0.8070197: 1 (15.4/4.1)

RES <= 0.7058755:

:...DM2 > -0.3974532:

:...HCRP1 > -0.2800881: 2 (8.7)

: HCRP1 <= -0.2800881:

: :...ESR2 <= -0.8700863: 2 (10/0.9)

: ESR2 > -0.8700863:

: :...RES <= -0.3740772: 1 (12.7/1.2)

: RES > -0.3740772: 2 (11.7/3.6)

DM2 <= -0.3974532:

:...PHD3 > -0.556608:

:...TEMP <= -0.8145545: 2 (14/0.4)

: TEMP > -0.8145545:

: :...EO2 <= -1.418525:

: :...NOH <= 0.7146246: 2 (11.2/2.7)

: : NOH > 0.7146246: 1 (3)

: EO2 > -1.418525:

: :...NEUT1 > -0.854023: 1 (12.8/1.6)

: NEUT1 <= -0.854023:

: :...LYM1 <= -1.111005: 1 (12/2.1)

: LYM1 > -1.111005: 2 (5.9/0.4)

PHD3 <= -0.556608:

:...LYM1 > -1.111005:

:...PCT1 > -0.9715286: 1 (46.3/5.4)

: PCT1 <= -0.9715286:

: :...PULSE <= -0.6376475: 1 (14.2)

: PULSE > -0.6376475:

: :...MONO1 <= -1.292171: 1 (10.8/2)

: MONO1 > -1.292171:

: :...AGE <= -1.372494: 1 (2.9)

: AGE > -1.372494: 2 (19.9/3.1)

LYM1 <= -1.111005:

:...MT1 > -0.3220414: 2 (8.6/1.4)

MT1 <= -0.3220414:

:...CRP2 <= -1.275555:

:...TEMP <= -0.6298622: 2 (2.1)

: TEMP > -0.6298622: 1 (14.9/2)

CRP2 > -1.275555:

:...PCT2 > -0.3162744: 1 (7.4/1.4)

PCT2 <= -0.3162744:

:...BRCH3 > -0.2330699: 1 (3/0.6)

BRCH3 <= -0.2330699:

:...MONO2 > -0.7270632: 2 (16.4/3.7)

MONO2 <= -0.7270632:

:...PCT1 <= -0.9715286: 2 (13.9/3.5)

PCT1 > -0.9715286: 1 (17.7/4.8)

----- Trial 10: -----

Decision tree:

ESR2 <= -0.8700863:

:...HCRP2 > -0.8173213:

: :...CD2 > -0.3974532: 2 (8.1)

: : CD2 <= -0.3974532:

: : :...CVD1 > -0.2025196: 2 (5.5)

: : CVD1 <= -0.2025196:

: : :...TEMP > 1.032368: 1 (4.4)

: : TEMP <= 1.032368:

: : :...DM2 > -0.3974532: 2 (5.2)

: : DM2 <= -0.3974532:

: : :...PHD3 <= -0.556608:

: : :...NOH <= -0.002178733: 1 (11.2/3.5)

: : : NOH > -0.002178733: 2 (10.8/1.4)

: : PHD3 > -0.556608:

: : :...RES <= 1.353847: 2 (10.4)

: : RES > 1.353847: 1 (2.1)

: HCRP2 <= -0.8173213:

: :...RES > 1.785828: 2 (8.4)

: RES <= 1.785828:

: :...ESR1 <= -0.8070197:

: :...SEX <= -2.204582: 2 (6.2)

: : SEX > -2.204582:

: : :...MT1 > -0.3220414: 2 (2.6)

: : MT1 <= -0.3220414:

: : :...EO1 <= -0.6598135: 2 (15.8/4.2)

: : EO1 > -0.6598135: 1 (4.6/0.1)

: ESR1 > -0.8070197:

: :...WBC1 > -1.189455:

: :...PCT2 > -0.3162744: 2 (2.5)

: : PCT2 <= -0.3162744:

: : :...PULSE <= -0.04893969: 1 (30.4/3.7)

: : PULSE > -0.04893969:

: : :...MONO1 <= -1.292171: 1 (4.4)

: : MONO1 > -1.292171:

: : :...CRP1 <= -0.556608: 1 (11.6/4.3)

: : CRP1 > -0.556608: 2 (6.3/0.1)

: WBC1 <= -1.189455:

: :...CVD1 > -0.2025196: 1 (2.2)

: CVD1 <= -0.2025196:

: :...NOH <= -0.7189821: 2 (7.2)

: NOH > -0.7189821:

: :...DP > 1.16914: 2 (2.7)

: DP <= 1.16914:

: :...NOH > -0.5397812: 2 (8.9/2.2)

: NOH <= -0.5397812:

: :...TEMP <= 0.4782911: 1 (12.1/0.4)

: TEMP > 0.4782911: 2 (2.3)

ESR2 > -0.8700863:

:...RES <= -1.238039: 2 (7.7/0.7)

RES > -1.238039:

:...CVD1 > -0.2025196: 1 (7.2/0.9)

CVD1 <= -0.2025196:

:...MT1 > -0.3220414:

:...EO1 <= -0.6598135: 1 (6.6/2.2)

: EO1 > -0.6598135: 2 (5.5/0.3)

MT1 <= -0.3220414:

:...HTN2 > -0.8381655: 1 (36.7/6)

HTN2 <= -0.8381655:

:...NOH > 1.610629: 2 (4.3)

NOH <= 1.610629:

:...TEMP > 1.955829: 1 (6.6)

TEMP <= 1.955829:

:...RES > -0.1580867:

:...EO1 > -0.6598135: 2 (9)

: EO1 <= -0.6598135:

: :...SP <= -0.5161568: 2 (8.1/0.9)

: SP > -0.5161568: 1 (9.1/0.7)

RES <= -0.1580867:

:...SEX <= -2.204582: 1 (4.7)

SEX > -2.204582:

:...AGE > 0.9715623: 2 (3.7)

AGE <= 0.9715623:

:...CRP2 <= -1.275555: 1 (2.4)

CRP2 > -1.275555:

:...SP <= -0.04757074: 1 (24.8/2.9)

SP > -0.04757074: 2 (6.4/0.5)

----- Trial 11: -----

Decision tree:

RES > 0.7058755:

:...HBV1 > -0.1472177: 2 (5.6)

: HBV1 <= -0.1472177:

: :...MONO1 <= -1.292171: 2 (16.1/1)

: MONO1 > -1.292171:

: :...CVD1 > -0.2025196: 2 (3.7)

: CVD1 <= -0.2025196:

: :...LYM1 <= -1.111005: 2 (11.9/1.7)

: LYM1 > -1.111005:

: :...PULSE <= 0.8929929: 1 (8)

: PULSE > 0.8929929: 2 (5.7)

RES <= 0.7058755:

:...HCRP2 > -0.8173213:

:...ESR1 > -0.8070197: 2 (33.6/6.8)

: ESR1 <= -0.8070197:

: :...EO1 > -0.6598135: 1 (13.6/2.5)

: EO1 <= -0.6598135:

: :...SEX <= -2.204582: 2 (10/0.6)

: SEX > -2.204582:

: :...SP <= 0.3272981: 1 (33.1/10.8)

: SP > 0.3272981: 2 (21.8/4.5)

HCRP2 <= -0.8173213:

:...MT1 > -0.3220414:

:...NOH <= -0.3605804: 1 (9.4/2.4)

: NOH > -0.3605804: 2 (13.1/1.6)

MT1 <= -0.3220414:

:...PULSE > 1.069605: 2 (15.8/3.2)

PULSE <= 1.069605:

:...SMK <= -1.67839:

:...EO1 > -0.6598135: 1 (6.1/0.9)

: EO1 <= -0.6598135:

: :...AGE <= -0.8397537: 1 (3.3)

: AGE > -0.8397537: 2 (18.9/4.7)

SMK > -1.67839:

:...HTN2 > -0.8381655: 1 (28.2/3)

HTN2 <= -0.8381655:

:...SP <= -2.43736: 2 (4)

SP > -2.43736:

:...BRCH3 > -0.2330699: 1 (3.4)

BRCH3 <= -0.2330699:

:...MONO2 <= -0.7270632:

:...SP <= 2.295359: 1 (31.9/6.4)

: SP > 2.295359: 2 (2)

MONO2 > -0.7270632:

:...ESR1 > -0.8070197: 2 (11.2/2.8)

ESR1 <= -0.8070197:

:...TEMP <= 0.2935989: 1 (14.9/2.4)

TEMP > 0.2935989: 2 (3.7/0.1)

----- Trial 12: -----

Decision tree:

NOH > 1.78983: 2 (17.7/1.8)

NOH <= 1.78983:

:...RES > 0.4898849:

:...CRP2 <= -1.275555: 2 (22.9/2.3)

: CRP2 > -1.275555:

: :...NOH <= -0.7189821: 2 (4.2)

: NOH > -0.7189821:

: :...NOH <= -0.5397812: 1 (9.5/0.9)

: NOH > -0.5397812: 2 (19.5/4.5)

RES <= 0.4898849:

:...PCT1 > -0.9715286:

:...LYM1 <= -1.111005:

: :...EO1 > -0.6598135: 1 (5.7/0.8)

: : EO1 <= -0.6598135:

: : :...DP > 1.247896: 1 (4)

: : DP <= 1.247896:

: : :...HCRP2 > -0.8173213: 2 (26.2/5)

: : HCRP2 <= -0.8173213:

: : :...CRP2 <= -1.275555: 2 (7.2/1.7)

: : CRP2 > -1.275555: 1 (7/1.6)

: LYM1 > -1.111005:

: :...PHD3 > -0.556608: 2 (9.5/4.6)

: PHD3 <= -0.556608:

: :...DP > -0.3272347: 1 (26.5)

: DP <= -0.3272347:

: :...CVD1 > -0.2025196: 2 (2.1)

: CVD1 <= -0.2025196:

: :...DM2 <= -0.3974532: 1 (22.2/3.3)

: DM2 > -0.3974532: 2 (4.8/1)

PCT1 <= -0.9715286:

:...MONO1 <= -1.292171:

:...PULSE > 1.776055: 2 (3.6)

: PULSE <= 1.776055:

: :...HTN2 > -0.8381655: 1 (8.6/0.1)

: HTN2 <= -0.8381655:

: :...MT1 > -0.3220414: 1 (3.5)

: MT1 <= -0.3220414:

: :...ESR1 <= -0.8070197: 1 (21.2/5)

: ESR1 > -0.8070197:

: :...TEMP <= 0.6629834: 2 (8.3)

: TEMP > 0.6629834: 1 (3.4)

MONO1 > -1.292171:

:...CRP2 <= -1.275555:

:...HCRP1 > -0.2800881: 2 (2.4)

: HCRP1 <= -0.2800881:

: :...TEMP <= -0.8145545: 2 (7.6/2.2)

: TEMP > -0.8145545: 1 (13.3/0.7)

CRP2 > -1.275555:

:...EO1 > -0.6598135: 2 (26.9/3.1)

EO1 <= -0.6598135:

:...PCT2 > -0.3162744: 1 (11/3.5)

PCT2 <= -0.3162744:

:...ESR2 <= -0.8700863: 2 (17.8/1.5)

ESR2 > -0.8700863:

:...TEMP <= -0.2604778: 2 (7.7/1)

TEMP > -0.2604778: 1 (5.1)

----- Trial 13: -----

Decision tree:

SMK <= -1.67839:

:...CKD1 > -0.1472177: 1 (5.3)

: CKD1 <= -0.1472177:

: :...LYM1 <= -1.111005:

: :...PULSE <= 1.363959: 2 (30.8/4.5)

: : PULSE > 1.363959: 1 (2.8)

: LYM1 > -1.111005:

: :...HCRP1 > -0.2800881: 2 (11.4/1.8)

: HCRP1 <= -0.2800881:

: :...PCT1 <= -0.9715286: 2 (13.6/3.5)

: PCT1 > -0.9715286: 1 (23.8/6.4)

SMK > -1.67839:

:...RES > 1.353847: 2 (17.9/3.8)

RES <= 1.353847:

:...MT1 > -0.3220414:

:...PCT1 <= -0.9715286: 2 (15.7/2.5)

: PCT1 > -0.9715286: 1 (6.6/1.4)

MT1 <= -0.3220414:

:...HTN2 > -0.8381655:

:...LYM2 <= -0.8487149: 1 (35.3/3)

: LYM2 > -0.8487149:

: :...BRCH3 > -0.2330699: 2 (4.5)

: BRCH3 <= -0.2330699:

: :...TEMP <= -0.2604778: 1 (11.8)

: TEMP > -0.2604778:

: :...PHD3 > -0.556608: 1 (3.5)

: PHD3 <= -0.556608:

: :...SP <= -0.844167: 1 (2.3)

: SP > -0.844167: 2 (9.7)

HTN2 <= -0.8381655:

:...NOH > 2.327432: 2 (7.2)

NOH <= 2.327432:

:...AGE <= -1.798686: 1 (8.7)

AGE > -1.798686:

:...DP > 0.7753571: 1 (24.7/4.1)

DP <= 0.7753571:

:...PCT1 > -0.9715286:

:...TEMP > 0.2935989: 1 (10.3/1)

: TEMP <= 0.2935989:

: :...NOH <= 0.3562229: 1 (13.3/4.1)

: NOH > 0.3562229: 2 (8)

PCT1 <= -0.9715286:

:...AGE <= -0.09391769:

:...PULSE <= -0.519906: 1 (7.6/2.6)

: PULSE > -0.519906: 2 (22.2/1)

AGE > -0.09391769:

:...PHD3 <= -0.556608: 1 (16.3/4.3)

PHD3 > -0.556608:

:...NOH <= -0.5397812: 1 (8.7/2.5)

NOH > -0.5397812: 2 (6.8)

----- Trial 14: -----

Decision tree:

SP > 1.545622: 2 (19.9/2.9)

SP <= 1.545622:

:...DM2 > -0.3974532:

:...CRP2 <= -1.275555: 2 (15.1/1.2)

: CRP2 > -1.275555:

: :...HTN2 <= -0.8381655: 2 (2.3)

: HTN2 > -0.8381655:

: :...CD2 <= -0.3974532: 1 (11.9/1.3)

: CD2 > -0.3974532: 2 (4.8/0.8)

DM2 <= -0.3974532:

:...LYM1 > -1.111005:

:...NOH > 1.073026: 2 (14.7)

: NOH <= 1.073026:

: :...AGE <= -1.372494: 1 (13.1)

: AGE > -1.372494:

: :...ESR2 > -0.8700863: 1 (40.7/8.7)

: ESR2 <= -0.8700863:

: :...NOH <= -0.7189821: 2 (8.1/1.3)

: NOH > -0.7189821:

: :...HCRP2 > -0.8173213:

: :...DP <= 0.4603309: 2 (14.7/3.2)

: : DP > 0.4603309: 1 (5.2)

: HCRP2 <= -0.8173213:

: :...HBV1 > -0.1472177: 2 (2.2)

: HBV1 <= -0.1472177:

: :...DP <= -1.744853: 2 (3.6)

: DP > -1.744853: 1 (34.6/4.6)

LYM1 <= -1.111005:

:...RES > 1.137856: 2 (9.9)

RES <= 1.137856:

:...LYM2 <= -0.8487149: 2 (9.3/2.1)

LYM2 > -0.8487149:

:...NOH > 0.7146246: 1 (10/1.4)

NOH <= 0.7146246:

:...TEMP > 0.1089067:

:...RES <= 0.2738944: 2 (25.3/2.2)

: RES > 0.2738944: 1 (8.3/2.7)

TEMP <= 0.1089067:

:...PCT2 > -0.3162744:

:...AGE <= 0.3322743: 1 (3.1)

: AGE > 0.3322743: 2 (9.2/0.6)

PCT2 <= -0.3162744:

:...CVD1 > -0.2025196: 1 (3.4)

CVD1 <= -0.2025196:

:...TEMP <= -0.8145545: 2 (14.7/2.8)

TEMP > -0.8145545:

:...SMK <= -1.67839: 1 (12.2/0.6)

SMK > -1.67839:

:...SP <= -1.219036: 2 (6.7/1)

SP > -1.219036: 1 (25.9/5.2)

----- Trial 15: -----

Decision tree:

NOH > 1.78983: 2 (17.4/1.9)

NOH <= 1.78983:

:...RES > 0.7058755:

:...MONO1 <= -1.292171: 2 (17.3/0.9)

: MONO1 > -1.292171:

: :...CVD1 > -0.2025196: 2 (2.6)

: CVD1 <= -0.2025196:

: :...PCT2 > -0.3162744: 2 (2.4)

: PCT2 <= -0.3162744:

: :...LYM1 <= -1.111005: 2 (5.8/1.2)

: LYM1 > -1.111005: 1 (13.8/3.8)

RES <= 0.7058755:

:...PCT1 > -0.9715286:

:...HCRP2 <= -0.8173213:

: :...PHD3 <= -0.556608: 1 (41.2/4.7)

: : PHD3 > -0.556608:

: : :...SMK <= -1.67839: 2 (5.5)

: : SMK > -1.67839:

: : :...BRCH3 <= -0.2330699: 1 (9.8/1.5)

: : BRCH3 > -0.2330699: 2 (2.6)

: HCRP2 > -0.8173213:

: :...SP > 1.170753: 2 (10.5/0.5)

: SP <= 1.170753:

: :...CKD1 > -0.1472177: 1 (2.6)

: CKD1 <= -0.1472177:

: :...SEX <= -2.204582: 2 (5.7/1.6)

: SEX > -2.204582:

: :...AGE > 0.4388223: 1 (10)

: AGE <= 0.4388223:

: :...CD2 > -0.3974532: 2 (3.1)

: CD2 <= -0.3974532:

: :...CVD1 > -0.2025196: 2 (2.2)

: CVD1 <= -0.2025196:

: :...RES <= -0.1580867: 1 (11.9/0.8)

: RES > -0.1580867:

: :...PULSE <= 0.1276727: 2 (6)

: PULSE > 0.1276727: 1 (6.9/1.5)

PCT1 <= -0.9715286:

:...ESR2 > -0.8700863:

:...MONO1 <= -1.292171: 1 (27.3/3.8)

: MONO1 > -1.292171:

: :...EO1 > -0.6598135: 2 (13.1/0.3)

: EO1 <= -0.6598135:

: :...NOH <= -0.1813796: 1 (18.8/3.9)

: NOH > -0.1813796: 2 (5.4/0.8)

ESR2 <= -0.8700863:

:...DM2 > -0.3974532: 2 (5.2)

DM2 <= -0.3974532:

:...HCRP2 > -0.8173213: 2 (16.3/1.9)

HCRP2 <= -0.8173213:

:...TEMP > 1.21706: 2 (7.2)

TEMP <= 1.21706:

:...EO1 > -0.6598135: 1 (17.6/5)

EO1 <= -0.6598135:

:...EO2 <= -1.418525: 2 (4.8)

EO2 > -1.418525:

:...PHD3 > -0.556608: 1 (4.7/0.7)

PHD3 <= -0.556608:

:...AGE <= -1.479042: 1 (3)

AGE > -1.479042:

:...NEUT1 <= -0.854023: 2 (13/1)

NEUT1 > -0.854023:

:...NOH <= -0.3605804: 1 (9.7/2.5)

NOH > -0.3605804: 2 (5.3)

----- Trial 16: -----

Decision tree:

HCRP1 > -0.2800881:

:...TEMP <= -0.6298622: 2 (17.9/0.1)

: TEMP > -0.6298622:

: :...DM2 <= -0.3974532: 1 (10.9/2.5)

: DM2 > -0.3974532: 2 (2.8)

HCRP1 <= -0.2800881:

:...ESR2 <= -0.8700863:

:...PCT1 <= -0.9715286:

: :...DP > 0.8541136:

: : :...CRP1 <= -0.556608: 1 (13.2/0.9)

: : : CRP1 > -0.556608: 2 (8/1.9)

: : DP <= 0.8541136:

: : :...CRP2 <= -1.275555:

: : :...RES <= -0.8060583: 1 (4.2/0.8)

: : : RES > -0.8060583: 2 (20.7/4.7)

: : CRP2 > -1.275555:

: : :...SP > -0.7973084: 2 (38.5/3.8)

: : SP <= -0.7973084:

: : :...TEMP <= -0.07578556: 2 (10.1/0.5)

: : TEMP > -0.07578556: 1 (6.2/0.6)

: PCT1 > -0.9715286:

: :...CD2 > -0.3974532: 2 (4.7)

: CD2 <= -0.3974532:

: :...MT1 > -0.3220414: 1 (4.6)

: MT1 <= -0.3220414:

: :...ESR1 <= -0.8070197:

: :...DP <= -0.1697216: 1 (4.3)

: : DP > -0.1697216: 2 (14.8/1.2)

: ESR1 > -0.8070197:

: :...HBV1 > -0.1472177: 1 (2.9)

: HBV1 <= -0.1472177:

: :...LYM1 > -1.111005: 1 (22.9/5.6)

: LYM1 <= -1.111005:

: :...PHD3 <= -0.556608: 1 (6.9/2)

: PHD3 > -0.556608: 2 (4.7)

ESR2 > -0.8700863:

:...CVD1 > -0.2025196: 1 (6.7/1.5)

CVD1 <= -0.2025196:

:...NOH > 1.610629: 2 (6.4)

NOH <= 1.610629:

:...AGE <= -0.4135617:

:...NOH > 0.7146246: 1 (3.8)

: NOH <= 0.7146246:

: :...LYM1 <= -1.111005:

: :...PULSE <= 0.009931089: 1 (4.3/0.8)

: : PULSE > 0.009931089: 2 (21.9/1.3)

: LYM1 > -1.111005:

: :...AGE <= -0.6266577: 1 (8.2)

: AGE > -0.6266577: 2 (8.5/0.3)

AGE > -0.4135617:

:...NEUT2 <= -1.104199: 1 (21.6/3.8)

NEUT2 > -1.104199:

:...CKD1 > -0.1472177: 1 (2.8)

CKD1 <= -0.1472177:

:...TEMP > 1.21706: 2 (6.4/0.4)

TEMP <= 1.21706:

:...PCT2 > -0.3162744: 1 (7.7/0.6)

PCT2 <= -0.3162744:

:...MT1 > -0.3220414: 2 (2.3/0.3)

MT1 <= -0.3220414:

:...NOH > -0.002178733: 2 (4.3/0.3)

NOH <= -0.002178733:

:...PULSE <= 1.069605: 1 (22.9/3.6)

PULSE > 1.069605: 2 (2.9)

----- Trial 17: -----

Decision tree:

RES > 0.7058755:

:...MT1 > -0.3220414: 1 (2.8/0.8)

: MT1 <= -0.3220414:

: :...MONO1 <= -1.292171: 2 (12.7)

: MONO1 > -1.292171:

: :...CVD1 > -0.2025196: 2 (4.3)

: CVD1 <= -0.2025196:

: :...HBV1 > -0.1472177: 2 (3.9)

: HBV1 <= -0.1472177:

: :...PULSE > 0.8341221: 2 (10.4)

: PULSE <= 0.8341221:

: :...LYM1 <= -1.111005: 2 (8.6/2.2)

: LYM1 > -1.111005: 1 (6.1)

RES <= 0.7058755:

:...DM2 > -0.3974532:

:...PCT2 > -0.3162744: 1 (4.3/0.6)

: PCT2 <= -0.3162744:

: :...PCT1 <= -0.9715286: 2 (7)

: PCT1 > -0.9715286:

: :...RES <= -0.3740772: 1 (10.3/2.1)

: RES > -0.3740772: 2 (18.3/2.3)

DM2 <= -0.3974532:

:...LYM1 > -1.111005:

:...NOH > 1.073026: 2 (9.6)

: NOH <= 1.073026:

: :...PCT2 > -0.3162744: 2 (9.2/3.2)

: PCT2 <= -0.3162744:

: :...PHD3 > -0.556608:

: :...RES <= 0.05790384: 2 (8/1)

: : RES > 0.05790384: 1 (4.2)

: PHD3 <= -0.556608:

: :...ESR2 > -0.8700863: 1 (24.6/2.7)

: ESR2 <= -0.8700863:

: :...PULSE <= 0.3631558: 1 (43.6/10.8)

: PULSE > 0.3631558: 2 (10/1.7)

LYM1 <= -1.111005:

:...PCT2 > -0.3162744:

:...AGE <= 0.7584663: 1 (11.4/1.8)

: AGE > 0.7584663: 2 (3.7)

PCT2 <= -0.3162744:

:...MT1 > -0.3220414:

:...SEX <= -2.204582: 1 (2.5)

: SEX > -2.204582: 2 (10.8/0.9)

MT1 <= -0.3220414:

:...SEX <= -2.204582: 2 (7.9/1.4)

SEX > -2.204582:

:...NOH > 1.073026: 1 (6.8/0.9)

NOH <= 1.073026:

:...PULSE > 1.187347: 2 (8)

PULSE <= 1.187347:

:...PULSE > 0.5397682: 1 (9.4/1)

PULSE <= 0.5397682:

:...MONO2 > -0.7270632:

:...ESR1 > -0.8070197: 2 (8.7)

: ESR1 <= -0.8070197:

: :...CRP2 <= -1.275555: 1 (3.5)

: CRP2 > -1.275555:

: :...NOH <= -0.3605804: 2 (10.1/1.2)

: NOH > -0.3605804: 1 (2.5)

MONO2 <= -0.7270632:

:...BRCH3 > -0.2330699: 2 (2.5/0.4)

BRCH3 <= -0.2330699:

:...PULSE > 0.1276727: 1 (8.9)

PULSE <= 0.1276727:

:...PULSE > 0.009931089: 2 (8.4)

PULSE <= 0.009931089:

:...ESR2 > -0.8700863: 1 (6.9)

ESR2 <= -0.8700863:

:...WBC1 <= -1.189455: 2 (10.3/1.1)

WBC1 > -1.189455: 1 (8.8/2.4)

----- Trial 18: -----

Decision tree:

RES > 1.137856: 2 (25.5/3.8)

RES <= 1.137856:

:...HCRP1 > -0.2800881: 2 (22.5/6.1)

HCRP1 <= -0.2800881:

:...HCRP2 > -0.8173213:

:...CD2 > -0.3974532: 2 (18.3/2.5)

: CD2 <= -0.3974532:

: :...MT1 > -0.3220414: 1 (4.9/0.7)

: MT1 <= -0.3220414:

: :...ESR1 > -0.8070197:

: :...PHD3 > -0.556608: 2 (6.4)

: : PHD3 <= -0.556608:

: : :...SP <= -0.7504498: 1 (5.4/0.5)

: : SP > -0.7504498: 2 (22.6/4.3)

: ESR1 <= -0.8070197:

: :...CKD1 > -0.1472177: 1 (2.9)

: CKD1 <= -0.1472177:

: :...DM2 > -0.3974532: 2 (14.5/3.8)

: DM2 <= -0.3974532:

: :...CRP1 > -0.556608: 2 (2)

: CRP1 <= -0.556608:

: :...AGE > 1.07811: 2 (6.2/0.5)

: AGE <= 1.07811:

: :...SP <= 0.93646: 1 (24.6/3.7)

: SP > 0.93646: 2 (4.8/0.5)

HCRP2 <= -0.8173213:

:...PCT1 > -0.9715286:

:...NOH <= 0.5354238: 1 (42.7/3.6)

: NOH > 0.5354238: 2 (8.3/0.8)

PCT1 <= -0.9715286:

:...SP <= -2.203067: 2 (7.5)

SP > -2.203067:

:...TEMP > 0.8476756: 2 (18/2.8)

TEMP <= 0.8476756:

:...TEMP > 0.6629834: 1 (6.9)

TEMP <= 0.6629834:

:...HTN2 > -0.8381655:

:...MT1 > -0.3220414: 2 (8.6)

: MT1 <= -0.3220414:

: :...SEX <= -2.204582: 2 (6.4/1)

: SEX > -2.204582: 1 (12.3/2)

HTN2 <= -0.8381655:

:...MT1 > -0.3220414: 1 (6.5)

MT1 <= -0.3220414:

:...TEMP <= -0.8145545: 2 (8.2)

TEMP > -0.8145545:

:...TEMP > 0.1089067: 2 (8.9/2)

TEMP <= 0.1089067:

:...SEX <= -2.204582: 1 (2.6)

SEX > -2.204582:

:...AGE <= 0.5453703: 1 (22.4/3)

AGE > 0.5453703: 2 (9/2.6)

----- Trial 19: -----

Decision tree:

LYM1 <= -1.111005:

:...RES > 1.137856: 2 (7.7)

: RES <= 1.137856:

: :...PCT2 > -0.3162744: 1 (15.9/4.8)

: PCT2 <= -0.3162744:

: :...HTN2 <= -0.8381655:

: :...SP <= -0.4224396: 2 (29.7/8.6)

: : SP > -0.4224396:

: : :...SP <= 0.6553083: 1 (27.8/3.1)

: : SP > 0.6553083:

: : :...EO1 <= -0.6598135: 2 (13.3/1.9)

: : EO1 > -0.6598135: 1 (7.2/1.8)

: HTN2 > -0.8381655:

: :...MT1 > -0.3220414: 2 (5.4)

: MT1 <= -0.3220414:

: :...BRCH3 > -0.2330699: 2 (4)

: BRCH3 <= -0.2330699:

: :...TEMP <= -0.2604778: 1 (8.3/0.9)

: TEMP > -0.2604778:

: :...MONO1 <= -1.292171: 2 (12.1)

: MONO1 > -1.292171:

: :...WBC1 <= -1.189455: 2 (7.1)

: WBC1 > -1.189455: 1 (8.8/2.3)

LYM1 > -1.111005:

:...NOH > 0.8938254: 2 (10.5)

NOH <= 0.8938254:

:...DM2 > -0.3974532:

:...HCRP1 > -0.2800881: 2 (3.8)

: HCRP1 <= -0.2800881:

: :...PCT1 <= -0.9715286: 2 (6/0.6)

: PCT1 > -0.9715286:

: :...RES <= 0.05790384: 1 (8.1)

: RES > 0.05790384: 2 (7.9/1.9)

DM2 <= -0.3974532:

:...AGE <= -1.372494: 1 (14.1)

AGE > -1.372494:

:...PULSE <= -1.402968: 1 (12.3/0.1)

PULSE > -1.402968:

:...SP <= -1.219036: 2 (9.3/0.5)

SP > -1.219036:

:...ESR2 <= -0.8700863:

:...PCT1 <= -0.9715286:

: :...PULSE <= -0.04893969: 1 (16.9/2.7)

: : PULSE > -0.04893969: 2 (17.9/1)

: PCT1 > -0.9715286:

: :...DP <= -0.4059912: 2 (13.1/3.5)

: DP > -0.4059912:

: :...SP <= 1.545622: 1 (19.5/1.1)

: SP > 1.545622: 2 (3.3/0.4)

ESR2 > -0.8700863:

:...CRP2 <= -1.275555: 1 (8.3)

CRP2 > -1.275555:

:...MT1 > -0.3220414: 2 (5.5/1.1)

MT1 <= -0.3220414:

:...PCT2 <= -0.3162744: 1 (20.4/2.3)

PCT2 > -0.3162744: 2 (4.6/1.3)

----- Trial 20: -----

Decision tree:

SEX <= -2.204582:

:...CKD1 > -0.1472177: 1 (4.5)

: CKD1 <= -0.1472177:

: :...SMK > -1.67839: 1 (5.2/0.9)

: SMK <= -1.67839:

: :...CD2 > -0.3974532: 2 (6.4/0.2)

: CD2 <= -0.3974532:

: :...MT1 > -0.3220414: 1 (2.3)

: MT1 <= -0.3220414:

: :...PCT1 <= -0.9715286: 2 (16.4/1.8)

: PCT1 > -0.9715286:

: :...PHD3 <= -0.556608: 1 (4.7/0.5)

: PHD3 > -0.556608: 2 (12.9/3.6)

SEX > -2.204582:

:...RES > 1.353847: 2 (19.3/4.8)

RES <= 1.353847:

:...LYM1 > -1.111005:

:...NOH > 1.073026: 2 (7.2)

: NOH <= 1.073026:

: :...BRCH3 > -0.2330699: 1 (7.3)

: BRCH3 <= -0.2330699:

: :...PCT1 <= -0.9715286:

: :...MONO1 <= -1.292171: 1 (20/1.8)

: : MONO1 > -1.292171:

: : :...PULSE <= -0.8731307: 1 (9.2/2.2)

: : PULSE > -0.8731307: 2 (31.4/5.2)

: PCT1 > -0.9715286:

: :...DP > -0.4059912: 1 (26.9/1.9)

: DP <= -0.4059912:

: :...SP <= -0.6567326: 1 (12.9)

: SP > -0.6567326:

: :...TEMP <= 0.8476756: 2 (9.5/1.3)

: TEMP > 0.8476756: 1 (4.6)

LYM1 <= -1.111005:

:...MT1 > -0.3220414: 2 (10.8/2.3)

MT1 <= -0.3220414:

:...HTN2 > -0.8381655:

:...AGE <= 0.4388223: 2 (25/5.4)

: AGE > 0.4388223: 1 (6.4/0.3)

HTN2 <= -0.8381655:

:...TEMP <= -0.6298622: 2 (9.7/1.1)

TEMP > -0.6298622:

:...CRP2 <= -1.275555: 1 (17/1.3)

CRP2 > -1.275555:

:...LYM2 <= -0.8487149: 1 (3.8)

LYM2 > -0.8487149:

:...SMK <= -1.67839: 1 (5.5/0.8)

SMK > -1.67839:

:...NOH > 0.7146246: 1 (3.7)

NOH <= 0.7146246:

:...HCRP2 > -0.8173213: 2 (12.7/2.5)

HCRP2 <= -0.8173213:

:...PULSE > 0.8341221: 2 (6.1)

PULSE <= 0.8341221:

:...RES <= -0.8060583: 2 (3.3)

RES > -0.8060583: 1 (24.4/5.2)

----- Trial 21: -----

Decision tree:

ESR2 > -0.8700863:

:...CD2 > -0.3974532:

: :...PULSE <= -0.4021644: 1 (8.2/0.7)

: : PULSE > -0.4021644: 2 (14.9/1.9)

: CD2 <= -0.3974532:

: :...CVD1 > -0.2025196: 1 (6.1)

: CVD1 <= -0.2025196:

: :...HTN2 > -0.8381655: 1 (37.6/7)

: HTN2 <= -0.8381655:

: :...EO1 > -0.6598135:

: :...CRP2 <= -1.275555: 1 (3.1/0.1)

: : CRP2 > -1.275555:

: : :...TEMP <= 1.955829: 2 (17.5/2.2)

: : TEMP > 1.955829: 1 (2.7)

: EO1 <= -0.6598135:

: :...SEX <= -2.204582: 1 (5.8/0.7)

: SEX > -2.204582:

: :...SP > 0.93646: 2 (3.8)

: SP <= 0.93646:

: :...NOH <= 1.610629: 1 (36.7/5.5)

: NOH > 1.610629: 2 (3)

ESR2 <= -0.8700863:

:...PCT1 > -0.9715286:

:...HBV1 > -0.1472177: 2 (7.8/1.6)

: HBV1 <= -0.1472177:

: :...WBC1 <= -1.189455:

: :...SP <= -0.4224396: 1 (6.9/0.3)

: : SP > -0.4224396: 2 (18.9/5)

: WBC1 > -1.189455:

: :...NOH <= 0.5354238: 1 (44.9/8.5)

: NOH > 0.5354238: 2 (5.1)

PCT1 <= -0.9715286:

:...DM2 > -0.3974532: 2 (6)

DM2 <= -0.3974532:

:...CD2 > -0.3974532: 1 (5.8/1.1)

CD2 <= -0.3974532:

:...TEMP > 1.401752: 2 (11.1)

TEMP <= 1.401752:

:...SEX <= -2.204582: 2 (11.5/1.8)

SEX > -2.204582:

:...MT1 > -0.3220414: 2 (5.9/1.3)

MT1 <= -0.3220414:

:...HTN2 > -0.8381655: 1 (11.8/2.9)

HTN2 <= -0.8381655:

:...TEMP <= -0.6298622: 2 (10.6)

TEMP > -0.6298622:

:...WBC1 > -1.189455: 1 (20.6/6.2)

WBC1 <= -1.189455:

:...LYM2 <= -0.8487149: 2 (9.3)

LYM2 > -0.8487149:

:...PHD3 <= -0.556608: 1 (6.6/0.2)

PHD3 > -0.556608: 2 (6.6/1.5)

----- Trial 22: -----

Decision tree:

LYM2 > -0.8487149:

:...RES > 1.137856: 2 (14.9)

: RES <= 1.137856:

: :...SEX <= -2.204582: 2 (17.7/4.3)

: SEX > -2.204582:

: :...BRCH3 > -0.2330699: 2 (7.2/1.4)

: BRCH3 <= -0.2330699:

: :...CVD1 > -0.2025196: 1 (6.2/0.8)

: CVD1 <= -0.2025196:

: :...MT1 > -0.3220414: 2 (8.4/1.5)

: MT1 <= -0.3220414:

: :...ESR1 <= -0.8070197:

: :...PULSE <= -0.1666813: 1 (20.2/1.6)

: : PULSE > -0.1666813:

: : :...NOH > 0.7146246: 1 (3.2)

: : NOH <= 0.7146246:

: : :...MONO1 <= -1.292171: 2 (10.6/1)

: : MONO1 > -1.292171:

: : :...NEUT1 <= -0.854023: 2 (13/2.7)

: : NEUT1 > -0.854023: 1 (8.6/2.1)

: ESR1 > -0.8070197:

: :...CD2 > -0.3974532: 2 (5.9)

: CD2 <= -0.3974532:

: :...TEMP > 1.21706: 2 (6.2)

: TEMP <= 1.21706:

: :...MONO1 <= -1.292171:

: :...PULSE <= 0.5397682: 2 (11.5)

: : PULSE > 0.5397682: 1 (2.7)

: MONO1 > -1.292171:

: :...TEMP > -0.07578556: 1 (8.9)

: TEMP <= -0.07578556:

: :...AGE <= -0.5201097: 2 (5.4)

: AGE > -0.5201097: 1 (11.6/3.7)

LYM2 <= -0.8487149:

:...DM2 > -0.3974532:

:...HCRP1 > -0.2800881: 2 (2.9)

: HCRP1 <= -0.2800881:

: :...DP <= -0.8785306: 2 (8)

: DP > -0.8785306: 1 (12.7/4.3)

DM2 <= -0.3974532:

:...AGE <= -1.372494: 1 (11.2)

AGE > -1.372494:

:...ESR2 > -0.8700863:

:...PHD3 <= -0.556608: 1 (40.7/8.1)

: PHD3 > -0.556608: 2 (8.2/2.3)

ESR2 <= -0.8700863:

:...NOH <= -0.7189821: 2 (9.3/1.1)

NOH > -0.7189821:

:...RES <= -1.238039: 1 (6.4)

RES > -1.238039:

:...HBV1 > -0.1472177: 2 (3.3)

HBV1 <= -0.1472177:

:...LYM1 <= -1.111005: 2 (3.1)

LYM1 > -1.111005:

:...NOH > 0.5354238: 2 (4.6)

NOH <= 0.5354238:

:...PULSE <= -0.519906: 1 (19.4/1.5)

PULSE > -0.519906:

:...SEX <= -2.204582: 2 (8.1/0.9)

SEX > -2.204582:

:...ESR1 <= -0.8070197: 1 (4.9)

ESR1 > -0.8070197:

:...PHD3 > -0.556608: 1 (2.4)

PHD3 <= -0.556608:

:...SMK <= -1.67839: 1 (2.2)

SMK > -1.67839: 2 (19.5/6.9)

----- Trial 23: -----

Decision tree:

HCRP2 <= -0.8173213:

:...BRCH3 > -0.2330699: 1 (11.1/1.7)

: BRCH3 <= -0.2330699:

: :...RES > 0.4898849:

: :...EO1 > -0.6598135: 2 (16.9/2.1)

: : EO1 <= -0.6598135:

: : :...SEX <= -2.204582: 2 (2.6)

: : SEX > -2.204582:

: : :...PCT1 <= -0.9715286: 2 (8.2/2)

: : PCT1 > -0.9715286: 1 (8.6/0.3)

: RES <= 0.4898849:

: :...PCT1 > -0.9715286:

: :...DP <= -1.744853: 2 (2.9)

: : DP > -1.744853: 1 (44.7/7.7)

: PCT1 <= -0.9715286:

: :...NEUT1 <= -0.854023:

: :...PCT2 > -0.3162744: 1 (7.1)

: : PCT2 <= -0.3162744:

: : :...HCRP1 > -0.2800881: 1 (5.6/0.8)

: : HCRP1 <= -0.2800881:

: : :...NOH <= -0.5397812: 1 (29.7/7.3)

: : NOH > -0.5397812: 2 (17.5/4.4)

: NEUT1 > -0.854023:

: :...HCRP1 > -0.2800881: 2 (2.7)

: HCRP1 <= -0.2800881:

: :...CRP1 > -0.556608: 1 (9.7/0.7)

: CRP1 <= -0.556608:

: :...SMK <= -1.67839: 1 (7.6/2)

: SMK > -1.67839: 2 (20.1/3.3)

HCRP2 > -0.8173213:

:...ESR2 <= -0.8700863: 2 (55.6/12.8)

ESR2 > -0.8700863:

:...CKD1 > -0.1472177: 1 (4.6)

CKD1 <= -0.1472177:

:...TEMP > 0.4782911: 2 (25.8/4.2)

TEMP <= 0.4782911:

:...PCT2 > -0.3162744: 1 (9)

PCT2 <= -0.3162744:

:...EO1 > -0.6598135: 1 (5.8)

EO1 <= -0.6598135:

:...PHD3 > -0.556608: 2 (6.3)

PHD3 <= -0.556608:

:...SEX <= -2.204582: 2 (2.2)

SEX > -2.204582:

:...PULSE <= 0.8929929: 1 (20.7/6.4)

PULSE > 0.8929929: 2 (4.3)

----- Trial 24: -----

Decision tree:

DM2 > -0.3974532:

:...PCT2 > -0.3162744: 1 (9/2.5)

: PCT2 <= -0.3162744:

: :...PCT1 <= -0.9715286: 2 (9.4)

: PCT1 > -0.9715286:

: :...TEMP <= -0.8145545: 1 (3.1)

: TEMP > -0.8145545: 2 (19.8/3.6)

DM2 <= -0.3974532:

:...LYM1 > -1.111005:

:...AGE > 0.9715623: 2 (26.2/7.5)

: AGE <= 0.9715623:

: :...SEX <= -2.204582:

: :...TEMP <= -0.2604778: 2 (8/1.2)

: : TEMP > -0.2604778: 1 (4.7)

: SEX > -2.204582:

: :...BRCH3 > -0.2330699: 1 (5.6)

: BRCH3 <= -0.2330699:

: :...HCRP1 > -0.2800881:

: :...EO1 <= -0.6598135: 1 (9.4/2.3)

: : EO1 > -0.6598135: 2 (3.5)

: HCRP1 <= -0.2800881:

: :...DP > 0.3815743: 1 (23.2)

: DP <= 0.3815743:

: :...PCT1 > -0.9715286:

: :...RES <= 0.2738944: 1 (22.6/0.9)

: : RES > 0.2738944: 2 (3.5/0.4)

: PCT1 <= -0.9715286:

: :...MONO1 <= -1.292171: 1 (10/1.2)

: MONO1 > -1.292171:

: :...PULSE <= -0.8731307: 1 (4.4)

: PULSE > -0.8731307:

: :...TEMP <= 1.586444: 2 (17.5/2.1)

: TEMP > 1.586444: 1 (2.4)

LYM1 <= -1.111005:

:...RES > 1.137856: 2 (9.1)

RES <= 1.137856:

:...CRP1 > -0.556608:

:...PULSE <= -0.4021644: 1 (3.6/0.1)

: PULSE > -0.4021644: 2 (15.4/2.3)

CRP1 <= -0.556608:

:...DP > 1.247896: 1 (8.7)

DP <= 1.247896:

:...HTN2 > -0.8381655:

:...TEMP > -0.07578556: 2 (13.9)

: TEMP <= -0.07578556:

: :...PCT1 <= -0.9715286: 2 (8.6/1.1)

: PCT1 > -0.9715286: 1 (6.9)

HTN2 <= -0.8381655:

:...SP > 0.6553083:

:...RES <= 0.2738944: 2 (14.2/1)

: RES > 0.2738944: 1 (2.9/0.6)

SP <= 0.6553083:

:...MT1 > -0.3220414: 1 (3.9)

MT1 <= -0.3220414:

:...LYM2 <= -0.8487149: 1 (3.7)

LYM2 > -0.8487149:

:...BRCH3 > -0.2330699: 1 (3.3)

BRCH3 <= -0.2330699:

:...NOH > -0.1813796: 1 (14.7/1.8)

NOH <= -0.1813796:

:...MONO1 <= -1.292171: 2 (9.3/1.9)

MONO1 > -1.292171:

:...PULSE > 0.6575097: 2 (3.9)

PULSE <= 0.6575097:

:...HCRP2 <= -0.8173213: 1 (12/2)

HCRP2 > -0.8173213: 2 (12.5/4.7)

----- Trial 25: -----

Decision tree:

HCRP1 > -0.2800881:

:...TEMP <= -0.6298622: 2 (11.8/0.5)

: TEMP > -0.6298622:

: :...DM2 <= -0.3974532: 1 (10.1/1.9)

: DM2 > -0.3974532: 2 (2.6)

HCRP1 <= -0.2800881:

:...LYM1 > -1.111005:

:...NOH > 0.7146246: 2 (9.1)

: NOH <= 0.7146246:

: :...PCT2 > -0.3162744:

: :...AGE <= -1.159398: 1 (2.4)

: : AGE > -1.159398: 2 (13.5/1.8)

: PCT2 <= -0.3162744:

: :...PULSE <= -0.6376475:

: :...NOH <= 0.3562229: 1 (39.5/0.9)

: : NOH > 0.3562229: 2 (2.4/0.3)

: PULSE > -0.6376475:

: :...SP > 1.077036: 2 (12.2/2.2)

: SP <= 1.077036:

: :...SEX <= -2.204582: 2 (9.5/4.3)

: SEX > -2.204582:

: :...CRP2 <= -1.275555: 1 (17.5)

: CRP2 > -1.275555:

: :...NOH <= -0.7189821: 2 (2.8)

: NOH > -0.7189821:

: :...PULSE <= -0.4610352: 2 (3.4)

: PULSE > -0.4610352: 1 (38.7/7.2)

LYM1 <= -1.111005:

:...PCT2 > -0.3162744:

:...DP <= -1.35107: 2 (2.8)

: DP > -1.35107: 1 (21.1/2.7)

PCT2 <= -0.3162744:

:...RES > 0.4898849: 2 (17.8/3.2)

RES <= 0.4898849:

:...CD2 > -0.3974532:

:...TEMP <= 0.2935989: 1 (3.4)

: TEMP > 0.2935989: 2 (8.4)

CD2 <= -0.3974532:

:...ESR2 > -0.8700863:

:...AGE <= -1.58559: 2 (3.9)

: AGE > -1.58559:

: :...MT1 > -0.3220414: 2 (4.1/1)

: MT1 <= -0.3220414:

: :...SP > 1.217612: 2 (3)

: SP <= 1.217612:

: :...SP <= -2.015632: 2 (2.6)

: SP > -2.015632: 1 (28.1/2.5)

ESR2 <= -0.8700863:

:...LYM2 <= -0.8487149: 2 (4.4)

LYM2 > -0.8487149:

:...TEMP > 1.21706: 2 (7.9)

TEMP <= 1.21706:

:...TEMP <= -0.8145545: 2 (9.2/0.8)

TEMP > -0.8145545:

:...BRCH3 > -0.2330699: 2 (2.2)

BRCH3 <= -0.2330699:

:...TEMP <= -0.2604778: 1 (9.7)

TEMP > -0.2604778:

:...HCRP2 <= -0.8173213: 1 (20.7/6.6)

HCRP2 > -0.8173213: 2 (4.3)

----- Trial 26: -----

Decision tree:

RES > 1.137856: 2 (27.5/5.9)

RES <= 1.137856:

:...ESR2 <= -0.8700863:

:...PCT1 > -0.9715286:

: :...CVD1 > -0.2025196: 2 (8.6/1.6)

: : CVD1 <= -0.2025196:

: : :...SMK > -1.67839: 1 (37.8/5.9)

: : SMK <= -1.67839:

: : :...MONO1 <= -1.292171: 2 (6)

: : MONO1 > -1.292171:

: : :...PHD3 <= -0.556608: 1 (9.5/1.5)

: : PHD3 > -0.556608: 2 (3.6)

: PCT1 <= -0.9715286:

: :...DM2 > -0.3974532: 2 (6.5)

: DM2 <= -0.3974532:

: :...CD2 > -0.3974532: 1 (7.6/1.5)

: CD2 <= -0.3974532:

: :...CRP1 > -0.556608:

: :...WBC1 <= -1.189455: 2 (5.1)

: : WBC1 > -1.189455:

: : :...PULSE <= 0.009931089: 1 (8.4)

: : PULSE > 0.009931089: 2 (5.7/1.7)

: CRP1 <= -0.556608:

: :...DP > 0.8541136: 1 (7.6/1)

: DP <= 0.8541136:

: :...WBC2 <= -0.7917082: 2 (31/4)

: WBC2 > -0.7917082:

: :...HCRP1 > -0.2800881: 1 (2.4)

: HCRP1 <= -0.2800881:

: :...PCT2 <= -0.3162744: 2 (20/4.6)

: PCT2 > -0.3162744: 1 (4.9/1.8)

ESR2 > -0.8700863:

:...DM2 > -0.3974532:

:...HTN2 <= -0.8381655: 2 (5)

: HTN2 > -0.8381655:

: :...PCT2 > -0.3162744: 1 (4)

: PCT2 <= -0.3162744:

: :...RES > -0.3740772: 2 (11.1/0.8)

: RES <= -0.3740772:

: :...PCT1 <= -0.9715286: 2 (2.9)

: PCT1 > -0.9715286: 1 (11.5/1)

DM2 <= -0.3974532:

:...CVD1 > -0.2025196: 1 (6.8)

CVD1 <= -0.2025196:

:...PHD3 > -0.556608:

:...LYM1 <= -1.111005: 1 (9.2/2.8)

: LYM1 > -1.111005: 2 (7.3/1.1)

PHD3 <= -0.556608:

:...CRP2 <= -1.275555: 1 (11.8)

CRP2 > -1.275555:

:...LYM2 <= -0.8487149: 1 (35.8/5)

LYM2 > -0.8487149:

:...PCT2 > -0.3162744: 1 (3.3)

PCT2 <= -0.3162744:

:...BRCH3 > -0.2330699: 1 (2)

BRCH3 <= -0.2330699:

:...PULSE <= -0.519906: 1 (7.5/1.4)

PULSE > -0.519906: 2 (18.4/3.7)

----- Trial 27: -----

Decision tree:

CD2 > -0.3974532:

:...MT1 > -0.3220414: 2 (8.5)

: MT1 <= -0.3220414:

: :...SMK <= -1.67839: 2 (12.6/1.4)

: SMK > -1.67839:

: :...CRP2 <= -1.275555: 1 (6.6/0.7)

: CRP2 > -1.275555:

: :...TEMP <= -0.44517: 1 (4.3)

: TEMP > -0.44517: 2 (13.8/3.7)

CD2 <= -0.3974532:

:...ESR2 <= -0.8700863:

:...ESR1 <= -0.8070197:

: :...SEX <= -2.204582: 2 (6.7)

: : SEX > -2.204582:

: : :...EO1 <= -0.6598135: 2 (22.6/4.1)

: : EO1 > -0.6598135:

: : :...MT1 <= -0.3220414: 1 (8.1/0.9)

: : MT1 > -0.3220414: 2 (3.4)

: ESR1 > -0.8070197:

: :...PULSE <= -1.402968: 1 (8.4)

: PULSE > -1.402968:

: :...HCRP2 > -0.8173213: 2 (28/7.8)

: HCRP2 <= -0.8173213:

: :...HCRP1 > -0.2800881:

: :...TEMP <= -0.6298622: 2 (8.7)

: : TEMP > -0.6298622: 1 (7.1/2.1)

: HCRP1 <= -0.2800881:

: :...TEMP > 1.21706: 2 (4.1)

: TEMP <= 1.21706:

: :...NOH > -0.3605804:

: :...PCT1 <= -0.9715286: 2 (10.2/0.8)

: : PCT1 > -0.9715286: 1 (9/2.1)

: NOH <= -0.3605804:

: :...WBC1 > -1.189455: 1 (28.4/3.1)

: WBC1 <= -1.189455:

: :...AGE <= -0.5201097: 2 (6.9)

: AGE > -0.5201097: 1 (14/2.8)

ESR2 > -0.8700863:

:...SEX <= -2.204582: 1 (20.6/3.1)

SEX > -2.204582:

:...CVD1 > -0.2025196: 1 (5.4)

CVD1 <= -0.2025196:

:...PULSE > 0.8341221:

:...SMK <= -1.67839: 1 (2.1)

: SMK > -1.67839: 2 (19.2/3.2)

PULSE <= 0.8341221:

:...AGE > 1.07811: 2 (6.7/1)

AGE <= 1.07811:

:...HTN2 > -0.8381655: 1 (22.8/2.4)

HTN2 <= -0.8381655:

:...DM2 > -0.3974532: 2 (3.5)

DM2 <= -0.3974532:

:...CRP2 <= -1.275555: 1 (5)

CRP2 > -1.275555:

:...SP <= 0.7958841: 1 (27.2/7.3)

SP > 0.7958841: 2 (5)

----- Trial 28: -----

Decision tree:

HCRP1 > -0.2800881:

:...EO1 > -0.6598135: 2 (7.6)

: EO1 <= -0.6598135:

: :...ESR2 > -0.8700863: 2 (3.6)

: ESR2 <= -0.8700863:

: :...NOH <= -0.3605804: 2 (7.7/2.2)

: NOH > -0.3605804: 1 (5.2)

HCRP1 <= -0.2800881:

:...PCT1 > -0.9715286:

:...NOH > 1.78983: 2 (9.1)

: NOH <= 1.78983:

: :...LYM1 > -1.111005:

: :...RES <= -0.1580867: 1 (34.3/0.4)

: : RES > -0.1580867:

: : :...CD2 > -0.3974532: 2 (3.3)

: : CD2 <= -0.3974532:

: : :...NOH <= -0.3605804: 1 (12.4/0.3)

: : NOH > -0.3605804:

: : :...PULSE <= 0.2454142: 1 (8.3/1.4)

: : PULSE > 0.2454142: 2 (10.3/1.1)

: LYM1 <= -1.111005:

: :...BRCH3 > -0.2330699: 2 (6.3/0.6)

: BRCH3 <= -0.2330699:

: :...SEX <= -2.204582: 2 (3.7/0.7)

: SEX > -2.204582:

: :...TEMP <= -0.07578556: 1 (14.4/1.3)

: TEMP > -0.07578556:

: :...EO1 > -0.6598135: 1 (6.6/0.4)

: EO1 <= -0.6598135:

: :...NOH <= -0.3605804: 2 (14.5/0.5)

: NOH > -0.3605804: 1 (10/2.9)

PCT1 <= -0.9715286:

:...RES > 1.353847: 2 (15.1/2.1)

RES <= 1.353847:

:...ESR2 > -0.8700863:

:...CRP2 <= -1.275555: 1 (7.6/0.3)

: CRP2 > -1.275555:

: :...EO1 > -0.6598135: 2 (25.4/4.6)

: EO1 <= -0.6598135:

: :...SMK <= -1.67839: 1 (6.9)

: SMK > -1.67839:

: :...RES <= -0.8060583: 2 (5.8/1.1)

: RES > -0.8060583: 1 (20.3/5)

ESR2 <= -0.8700863:

:...EO1 > -0.6598135:

:...CD2 > -0.3974532: 1 (4.2)

: CD2 <= -0.3974532:

: :...SP <= -0.609874: 1 (7/0.1)

: SP > -0.609874:

: :...NOH <= 0.8938254: 2 (17.9/3.3)

: NOH > 0.8938254: 1 (2.3)

EO1 <= -0.6598135:

:...AGE <= -1.265946: 1 (4.3/0.2)

AGE > -1.265946:

:...CD2 > -0.3974532: 2 (5.9)

CD2 <= -0.3974532:

:...TEMP > 0.2935989: 1 (8/2.6)

TEMP <= 0.2935989:

:...ESR1 <= -0.8070197: 2 (4.4)

ESR1 > -0.8070197:

:...LYM1 > -1.111005: 2 (14.8/2)

LYM1 <= -1.111005:

:...CRP1 > -0.556608: 2 (5.1)

CRP1 <= -0.556608:

:...DP <= -0.7210174: 2 (5.3)

DP > -0.7210174: 1 (11.5/2.2)

----- Trial 29: -----

Decision tree:

DM2 > -0.3974532:

:...HCRP1 > -0.2800881: 2 (6.4)

: HCRP1 <= -0.2800881:

: :...RES <= -0.1580867: 1 (9.9/2.9)

: RES > -0.1580867: 2 (20/3.4)

DM2 <= -0.3974532:

:...LYM1 <= -1.111005:

:...RES > 1.137856: 2 (13.7)

: RES <= 1.137856:

: :...TEMP <= 0.1089067:

: :...TEMP <= -0.8145545:

: : :...CVD1 <= -0.2025196: 2 (16.9/2.7)

: : : CVD1 > -0.2025196: 1 (3.6)

: : TEMP > -0.8145545:

: : :...PULSE > 0.3631558:

: : :...TEMP <= -0.44517: 1 (4.4/0.7)

: : : TEMP > -0.44517: 2 (10.7/1.5)

: : PULSE <= 0.3631558:

: : :...MONO1 > -1.292171: 1 (29.3/3.5)

: : MONO1 <= -1.292171:

: : :...TEMP <= -0.44517: 1 (7.2)

: : TEMP > -0.44517: 2 (12.2/4)

: TEMP > 0.1089067:

: :...NOH > 0.8938254: 1 (3.4)

: NOH <= 0.8938254:

: :...DP > 1.247896: 1 (3.5)

: DP <= 1.247896:

: :...HCRP2 > -0.8173213: 2 (15.1)

: HCRP2 <= -0.8173213:

: :...CD2 > -0.3974532: 1 (2)

: CD2 <= -0.3974532:

: :...AGE <= 0.6519183: 2 (28.1/4.9)

: AGE > 0.6519183: 1 (2.8/0.5)

LYM1 > -1.111005:

:...AGE <= -1.372494: 1 (12.3)

AGE > -1.372494:

:...NOH > 0.5354238: 2 (11.2/2.3)

NOH <= 0.5354238:

:...SP <= -1.219036: 2 (8.3/1.1)

SP > -1.219036:

:...SP > 1.545622: 2 (5.5/0.2)

SP <= 1.545622:

:...PCT2 > -0.3162744: 2 (10.4/4.7)

PCT2 <= -0.3162744:

:...NEUT1 > -0.854023: 1 (45.4/7.7)

NEUT1 <= -0.854023:

:...ESR1 <= -0.8070197: 1 (25/4)

ESR1 > -0.8070197:

:...SP <= -0.1881466: 1 (9.8/1.3)

SP > -0.1881466: 2 (11.9/0.6)

----- Trial 30: -----

Decision tree:

RES > 0.7058755:

:...MONO1 <= -1.292171: 2 (16.7/0.5)

: MONO1 > -1.292171:

: :...LYM1 <= -1.111005: 2 (10.9/1.1)

: LYM1 > -1.111005:

: :...PULSE <= 0.8929929: 1 (10.4/2.2)

: PULSE > 0.8929929: 2 (5.3)

RES <= 0.7058755:

:...DM2 > -0.3974532:

:...PCT2 > -0.3162744: 1 (6.5/1.4)

: PCT2 <= -0.3162744:

: :...PCT1 <= -0.9715286: 2 (13.9)

: PCT1 > -0.9715286:

: :...CRP2 <= -1.275555: 2 (8.5/1.5)

: CRP2 > -1.275555: 1 (11.3/3.6)

DM2 <= -0.3974532:

:...HCRP1 > -0.2800881: 1 (14.1/2.7)

HCRP1 <= -0.2800881:

:...ESR2 <= -0.8700863:

:...EO1 > -0.6598135:

: :...PULSE > 2.070409: 2 (3.1)

: : PULSE <= 2.070409:

: : :...HCRP2 <= -0.8173213: 1 (31/5.6)

: : HCRP2 > -0.8173213:

: : :...SP <= -0.609874: 1 (3.5)

: : SP > -0.609874: 2 (9.2/1.1)

: EO1 <= -0.6598135:

: :...EO2 <= -1.418525: 2 (5.2)

: EO2 > -1.418525:

: :...PULSE <= -0.8731307: 1 (19.9/4.3)

: PULSE > -0.8731307:

: :...CRP2 > -1.275555: 2 (43.8/9.9)

: CRP2 <= -1.275555:

: :...PULSE <= 0.009931089: 1 (8.6/1.1)

: PULSE > 0.009931089: 2 (11.6/2.4)

ESR2 > -0.8700863:

:...CVD1 > -0.2025196: 1 (5)

CVD1 <= -0.2025196:

:...MT1 > -0.3220414:

:...RES <= -0.5900677: 2 (8.7/0.7)

: RES > -0.5900677: 1 (3.1)

MT1 <= -0.3220414:

:...PHD3 > -0.556608:

:...MONO1 <= -1.292171: 1 (9.3/1.9)

: MONO1 > -1.292171: 2 (9.1/2.3)

PHD3 <= -0.556608:

:...BRCH3 > -0.2330699: 1 (4.3)

BRCH3 <= -0.2330699:

:...CRP2 <= -1.275555: 1 (2.9)

CRP2 > -1.275555:

:...LYM2 <= -0.8487149: 1 (27.5/3.8)

LYM2 > -0.8487149:

:...PCT2 > -0.3162744: 1 (3.3)

PCT2 <= -0.3162744:

:...RES > 0.2738944: 1 (4.2)

RES <= 0.2738944:

:...PULSE <= -0.8142599: 1 (3.3)

PULSE > -0.8142599: 2 (14.8/4.5)

----- Trial 31: -----

Decision tree:

NOH > 1.252227:

:...AGE <= -0.7332057: 1 (2.9)

: AGE > -0.7332057: 2 (17.7/0.8)

NOH <= 1.252227:

:...RES > 0.7058755:

:...HBV1 > -0.1472177: 2 (5.4)

: HBV1 <= -0.1472177:

: :...CVD1 > -0.2025196: 2 (2.8)

: CVD1 <= -0.2025196:

: :...MONO1 <= -1.292171: 2 (12.3/1.3)

: MONO1 > -1.292171:

: :...RES > 2.4338: 2 (4.6)

: RES <= 2.4338:

: :...PULSE <= 0.8929929: 1 (9.9/1.3)

: PULSE > 0.8929929: 2 (4.6)

RES <= 0.7058755:

:...DM2 > -0.3974532:

:...HCRP1 > -0.2800881: 2 (4)

: HCRP1 <= -0.2800881:

: :...HCRP2 > -0.8173213: 2 (20.7/4.8)

: HCRP2 <= -0.8173213:

: :...AGE <= 0.9715623: 1 (10.8/1.2)

: AGE > 0.9715623: 2 (3.9)

DM2 <= -0.3974532:

:...PCT1 > -0.9715286:

:...BRCH3 > -0.2330699:

: :...HTN2 <= -0.8381655: 1 (3.1)

: : HTN2 > -0.8381655: 2 (9/1.9)

: BRCH3 <= -0.2330699:

: :...SMK > -1.67839: 1 (50.9/8.4)

: SMK <= -1.67839:

: :...PHD3 > -0.556608: 2 (5)

: PHD3 <= -0.556608:

: :...MONO1 <= -1.292171: 2 (4.3)

: MONO1 > -1.292171: 1 (15.7/1.3)

PCT1 <= -0.9715286:

:...TEMP > 1.21706: 2 (14.7/1.7)

TEMP <= 1.21706:

:...ESR2 > -0.8700863:

:...MONO1 <= -1.292171: 1 (22.5/2.2)

: MONO1 > -1.292171:

: :...EO1 > -0.6598135: 2 (6/0.4)

: EO1 <= -0.6598135:

: :...NEUT1 > -0.854023: 1 (2.7)

: NEUT1 <= -0.854023:

: :...SP <= -0.5161568: 1 (6.5)

: SP > -0.5161568: 2 (10.8/3.3)

ESR2 <= -0.8700863:

:...HCRP2 > -0.8173213: 2 (15.3/2.4)

HCRP2 <= -0.8173213:

:...CD2 > -0.3974532: 1 (3.6)

CD2 <= -0.3974532:

:...RES > 0.4898849: 2 (4.7)

RES <= 0.4898849:

:...TEMP <= -0.8145545: 2 (12.3/2.4)

TEMP > -0.8145545:

:...HCRP1 > -0.2800881: 1 (2.4)

HCRP1 <= -0.2800881:

:...NOH > -0.3605804: 2 (7.4/0.9)

NOH <= -0.3605804:

:...NEUT1 > -0.854023: 1 (12.7)

NEUT1 <= -0.854023:

:...PULSE <= -0.04893969: 2 (4.8)

PULSE > -0.04893969: 1 (15/3.8)

----- Trial 32: -----

Decision tree:

RES > 0.7058755:

:...MT1 <= -0.3220414: 2 (36.2/6.7)

: MT1 > -0.3220414: 1 (3.1/0.3)

RES <= 0.7058755:

:...CD2 > -0.3974532:

:...TEMP <= -0.6298622: 1 (5)

: TEMP > -0.6298622: 2 (23.4/5.2)

CD2 <= -0.3974532:

:...ESR2 <= -0.8700863:

:...SEX <= -2.204582: 2 (19.4/5.3)

: SEX > -2.204582:

: :...TEMP > 1.401752: 2 (7/0.2)

: TEMP <= 1.401752:

: :...AGE > 1.07811: 2 (9.3/1.4)

: AGE <= 1.07811:

: :...LYM1 <= -1.111005:

: :...MONO1 <= -1.292171:

: : :...AGE <= -1.905234: 1 (3.3)

: : : AGE > -1.905234: 2 (17.5/2.9)

: : MONO1 > -1.292171:

: : :...AGE <= -0.5201097: 2 (15.2/3)

: : AGE > -0.5201097: 1 (22.9/3.5)

: LYM1 > -1.111005:

: :...DP > 0.3815743: 1 (17.1)

: DP <= 0.3815743:

: :...RES > 0.2738944: 2 (4.5/0.2)

: RES <= 0.2738944:

: :...CRP2 <= -1.275555: 1 (15.2/1.5)

: CRP2 > -1.275555: 2 (12.4/3.4)

ESR2 > -0.8700863:

:...NOH > 1.610629: 2 (7.2)

NOH <= 1.610629:

:...CKD1 > -0.1472177: 1 (7.9)

CKD1 <= -0.1472177:

:...LYM2 <= -0.8487149:

:...MONO1 <= -1.292171: 1 (20.2)

: MONO1 > -1.292171:

: :...PCT2 <= -0.3162744: 1 (23.9/6.3)

: PCT2 > -0.3162744: 2 (8.1/1.3)

LYM2 > -0.8487149:

:...AGE <= -1.58559: 2 (6.4)

AGE > -1.58559:

:...PCT2 > -0.3162744: 1 (11.1/1)

PCT2 <= -0.3162744:

:...SP > 1.217612: 2 (5.3)

SP <= 1.217612:

:...PCT1 > -0.9715286: 1 (15.3/2.6)

PCT1 <= -0.9715286:

:...NOH <= -0.5397812: 1 (6.5/1)

NOH > -0.5397812: 2 (5.5)

----- Trial 33: -----

Decision tree:

DM2 > -0.3974532:

:...HTN2 <= -0.8381655: 2 (5.8/0.9)

: HTN2 > -0.8381655:

: :...PCT2 > -0.3162744: 1 (5.4/0.2)

: PCT2 <= -0.3162744:

: :...PCT1 <= -0.9715286: 2 (8.6)

: PCT1 > -0.9715286:

: :...RES <= -0.3740772: 1 (9.1/0.5)

: RES > -0.3740772:

: :...TEMP <= -0.8145545: 1 (2.7)

: TEMP > -0.8145545: 2 (14.6/0.8)

DM2 <= -0.3974532:

:...PHD3 > -0.556608:

:...TEMP <= -0.8145545: 2 (17.2/1.1)

: TEMP > -0.8145545:

: :...DP > 0.3815743: 1 (17.1/1.3)

: DP <= 0.3815743:

: :...LYM1 > -1.111005: 2 (9.8/1)

: LYM1 <= -1.111005:

: :...CRP1 > -0.556608: 2 (3.9/0.6)

: CRP1 <= -0.556608:

: :...PCT2 <= -0.3162744: 1 (10.8/1.6)

: PCT2 > -0.3162744: 2 (7.5/2.1)

PHD3 <= -0.556608:

:...LYM2 <= -0.8487149:

:...AGE <= -1.372494: 1 (14.1)

: AGE > -1.372494:

: :...NOH <= -0.7189821:

: :...ESR2 <= -0.8700863: 2 (11.1/1.5)

: : ESR2 > -0.8700863: 1 (2.7)

: NOH > -0.7189821:

: :...MT1 > -0.3220414:

: :...NOH <= -0.3605804: 1 (4.5)

: : NOH > -0.3605804: 2 (8.2/1.3)

: MT1 <= -0.3220414:

: :...WBC2 <= -0.7917082:

: :...HBV1 > -0.1472177: 2 (2.7/0.5)

: : HBV1 <= -0.1472177:

: : :...NOH <= 0.5354238: 1 (43.4/4.1)

: : NOH > 0.5354238: 2 (2.6)

: WBC2 > -0.7917082:

: :...CRP1 > -0.556608: 1 (7.1)

: CRP1 <= -0.556608:

: :...TEMP <= 0.4782911: 1 (15.8/5.2)

: TEMP > 0.4782911: 2 (6.6/0.5)

LYM2 > -0.8487149:

:...TEMP > 1.21706: 2 (9.4)

TEMP <= 1.21706:

:...HCRP2 > -0.8173213:

:...PCT2 > -0.3162744: 1 (4.4)

: PCT2 <= -0.3162744:

: :...DP <= -1.193557: 1 (2.9/0.1)

: DP > -1.193557: 2 (28.2/4.9)

HCRP2 <= -0.8173213:

:...PULSE > 1.246217: 2 (3.3)

PULSE <= 1.246217:

:...PCT1 > -0.9715286: 1 (13.5/0.4)

PCT1 <= -0.9715286:

:...ESR2 <= -0.8700863:

:...TEMP <= -0.8145545: 2 (2.7)

: TEMP > -0.8145545: 1 (21.4/3.6)

ESR2 > -0.8700863:

:...DP <= 0.3815743: 2 (9.3/1.5)

DP > 0.3815743: 1 (2.5)

----- Trial 34: -----

Decision tree:

RES > 1.137856:

:...LYM1 <= -1.111005: 2 (13.9)

: LYM1 > -1.111005:

: :...PHD3 <= -0.556608: 2 (15.4/3.4)

: PHD3 > -0.556608: 1 (3.1)

RES <= 1.137856:

:...AGE > 1.07811:

:...SP <= -1.40647: 1 (2.7)

: SP > -1.40647:

: :...NOH > -0.3605804: 2 (13.9)

: NOH <= -0.3605804:

: :...TEMP <= -0.44517: 1 (10.6/2.4)

: TEMP > -0.44517: 2 (9.2/0.5)

AGE <= 1.07811:

:...SEX <= -2.204582:

:...CKD1 > -0.1472177: 1 (3.8)

: CKD1 <= -0.1472177:

: :...PHD3 > -0.556608: 2 (7)

: PHD3 <= -0.556608:

: :...PCT1 <= -0.9715286: 2 (11.4/1.2)

: PCT1 > -0.9715286:

: :...DM2 <= -0.3974532: 1 (9.7)

: DM2 > -0.3974532: 2 (6.1/1)

SEX > -2.204582:

:...ESR2 <= -0.8700863:

:...LYM1 <= -1.111005:

: :...CRP2 <= -1.275555:

: : :...WBC1 <= -1.189455: 2 (10.2/2.6)

: : : WBC1 > -1.189455: 1 (17.7/3)

: : CRP2 > -1.275555:

: : :...WBC1 > -1.189455:

: : :...DP <= 1.326653: 2 (20.7/1.3)

: : : DP > 1.326653: 1 (2.7)

: : WBC1 <= -1.189455:

: : :...PCT1 > -0.9715286: 1 (4.7)

: : PCT1 <= -0.9715286:

: : :...DP <= 0.3028178: 2 (10.9/2.7)

: : DP > 0.3028178: 1 (5.1)

: LYM1 > -1.111005:

: :...BRCH3 > -0.2330699: 1 (4.4)

: BRCH3 <= -0.2330699:

: :...CKD1 > -0.1472177: 2 (2.8)

: CKD1 <= -0.1472177:

: :...NEUT1 > -0.854023: 1 (33.2/4.2)

: NEUT1 <= -0.854023:

: :...EO1 <= -0.6598135: 2 (15.7/3.8)

: EO1 > -0.6598135: 1 (6.3/0.9)

ESR2 > -0.8700863:

:...CVD1 > -0.2025196: 1 (4.8)

CVD1 <= -0.2025196:

:...DM2 > -0.3974532: 2 (8.2/3.2)

DM2 <= -0.3974532:

:...RES <= -0.8060583: 2 (8/2.8)

RES > -0.8060583:

:...SMK <= -1.67839: 1 (5.9)

SMK > -1.67839:

:...EO1 <= -0.6598135: 1 (46.6/8.4)

EO1 > -0.6598135:

:...HCRP2 <= -0.8173213: 2 (9.6/3.3)

HCRP2 > -0.8173213: 1 (4.9)

----- Trial 35: -----

Decision tree:

RES > 0.4898849:

:...CRP2 <= -1.275555: 2 (27.9/2.8)

: CRP2 > -1.275555:

: :...SEX <= -2.204582: 2 (4.7)

: SEX > -2.204582:

: :...RES > 2.217809: 2 (4.7)

: RES <= 2.217809:

: :...DP > 0.3815743: 1 (11.2/0.8)

: DP <= 0.3815743:

: :...SP <= -0.9378842: 1 (5.4/0.3)

: SP > -0.9378842: 2 (11.6/1.1)

RES <= 0.4898849:

:...PHD3 > -0.556608:

:...NEUT1 > -0.854023: 2 (15.5/2.7)

: NEUT1 <= -0.854023:

: :...CRP2 > -1.275555: 1 (20.6/4.3)

: CRP2 <= -1.275555:

: :...DP <= -0.6422609: 1 (5.4/0.8)

: DP > -0.6422609: 2 (12.3/1.2)

PHD3 <= -0.556608:

:...PCT1 <= -0.9715286:

:...MONO2 > -0.7270632:

: :...HTN2 > -0.8381655: 1 (8.3)

: : HTN2 <= -0.8381655:

: : :...CRP1 > -0.556608: 1 (4.6)

: : CRP1 <= -0.556608:

: : :...MT1 > -0.3220414: 1 (4.1/0.5)

: : MT1 <= -0.3220414:

: : :...ESR1 <= -0.8070197: 1 (9.2/2.6)

: : ESR1 > -0.8070197: 2 (5.3)

: MONO2 <= -0.7270632:

: :...MT1 > -0.3220414:

: :...NOH <= -0.3605804: 1 (4.5/1.2)

: : NOH > -0.3605804: 2 (7.6)

: MT1 <= -0.3220414:

: :...SMK <= -1.67839: 2 (17/3.9)

: SMK > -1.67839:

: :...ESR1 <= -0.8070197:

: :...CRP2 <= -1.275555: 1 (9.2)

: : CRP2 > -1.275555:

: : :...PULSE <= -0.4021644: 1 (6.5)

: : PULSE > -0.4021644: 2 (5.3/1.3)

: ESR1 > -0.8070197:

: :...NEUT1 <= -0.854023: 2 (6.8)

: NEUT1 > -0.854023:

: :...PULSE <= 0.009931089: 1 (11.5/1.1)

: PULSE > 0.009931089: 2 (6/0.7)

PCT1 > -0.9715286:

:...HCRP2 <= -0.8173213: 1 (53.7/6.6)

HCRP2 > -0.8173213:

:...CD2 > -0.3974532: 2 (7/1.1)

CD2 <= -0.3974532:

:...RES <= -0.5900677: 1 (11.3/0.4)

RES > -0.5900677:

:...NOH > -0.1813796: 2 (7.3)

NOH <= -0.1813796:

:...DM2 > -0.3974532: 2 (3)

DM2 <= -0.3974532:

:...SP > 1.26447: 2 (3.9)

SP <= 1.26447:

:...SP <= -1.265894: 2 (2.6)

SP > -1.265894: 1 (14.9)

----- Trial 36: -----

Decision tree:

NOH > 1.78983:

:...AGE <= -0.7332057: 1 (2.8)

: AGE > -0.7332057: 2 (16.3)

NOH <= 1.78983:

:...PCT1 > -0.9715286:

:...SMK <= -1.67839:

: :...MONO1 <= -1.292171: 2 (16.9/0.8)

: : MONO1 > -1.292171:

: : :...SP <= 0.8427427: 1 (26/5.1)

: : SP > 0.8427427: 2 (5.6/0.6)

: SMK > -1.67839:

: :...RES > 1.353847: 2 (6.1/0.8)

: RES <= 1.353847:

: :...HCRP2 <= -0.8173213:

: :...NOH <= 0.5354238: 1 (43.5/0.3)

: : NOH > 0.5354238: 2 (6.2/1.6)

: HCRP2 > -0.8173213:

: :...CD2 > -0.3974532: 2 (3.1)

: CD2 <= -0.3974532:

: :...CRP1 > -0.556608: 2 (4.6/1)

: CRP1 <= -0.556608:

: :...AGE > -0.5201097: 1 (14.1)

: AGE <= -0.5201097:

: :...PHD3 > -0.556608: 1 (4.5)

: PHD3 <= -0.556608:

: :...RES <= -0.3740772: 1 (3.3)

: RES > -0.3740772: 2 (8.8/0.2)

PCT1 <= -0.9715286:

:...ESR2 > -0.8700863:

:...CRP2 <= -1.275555:

: :...NOH <= -0.1813796: 1 (11.5)

: : NOH > -0.1813796: 2 (2.4)

: CRP2 > -1.275555:

: :...EO1 > -0.6598135: 2 (16.9/4.4)

: EO1 <= -0.6598135:

: :...EO2 <= -1.418525: 1 (4.6)

: EO2 > -1.418525:

: :...SMK <= -1.67839: 1 (7.2/0.8)

: SMK > -1.67839:

: :...PCT2 <= -0.3162744: 2 (15.3/4.6)

: PCT2 > -0.3162744: 1 (9.5/2.1)

ESR2 <= -0.8700863:

:...DM2 > -0.3974532: 2 (7)

DM2 <= -0.3974532:

:...CD2 > -0.3974532: 1 (5/0.9)

CD2 <= -0.3974532:

:...TEMP <= -0.8145545: 2 (23.1/2.1)

TEMP > -0.8145545:

:...TEMP > 1.401752: 2 (9.2)

TEMP <= 1.401752:

:...NEUT1 > -0.854023: 1 (21.2/5)

NEUT1 <= -0.854023:

:...HCRP1 > -0.2800881: 1 (2.2)

HCRP1 <= -0.2800881:

:...HCRP2 > -0.8173213: 2 (6.8)

HCRP2 <= -0.8173213:

:...LYM2 <= -0.8487149: 2 (8.6/0.9)

LYM2 > -0.8487149:

:...MONO1 <= -1.292171: 2 (4.5/0.7)

MONO1 > -1.292171: 1 (12.4/2.6)

----- Trial 37: -----

Decision tree:

HCRP1 > -0.2800881:

:...EO1 > -0.6598135: 2 (8.2)

: EO1 <= -0.6598135:

: :...DM2 > -0.3974532: 2 (4.9)

: DM2 <= -0.3974532:

: :...TEMP <= -0.6298622: 2 (5.2/0.4)

: TEMP > -0.6298622: 1 (9/0.9)

HCRP1 <= -0.2800881:

:...LYM1 > -1.111005:

:...NOH > 0.7146246: 2 (14.8)

: NOH <= 0.7146246:

: :...AGE <= 0.7584663: 1 (111.7/20.5)

: AGE > 0.7584663: 2 (25.5/9)

LYM1 <= -1.111005:

:...RES > 1.137856: 2 (14.2)

RES <= 1.137856:

:...ESR2 <= -0.8700863:

:...LYM2 <= -0.8487149: 2 (3.8)

: LYM2 > -0.8487149:

: :...TEMP > 1.401752: 2 (7.1)

: TEMP <= 1.401752:

: :...TEMP > 0.6629834: 1 (5.8)

: TEMP <= 0.6629834:

: :...EO1 <= -0.6598135: 2 (47.2/13)

: EO1 > -0.6598135:

: :...CRP2 <= -1.275555: 1 (8.5/1.1)

: CRP2 > -1.275555: 2 (8.1/2.6)

ESR2 > -0.8700863:

:...AGE <= -1.58559: 2 (4.1)

AGE > -1.58559:

:...TEMP <= -0.44517: 1 (15.1/1.2)

TEMP > -0.44517:

:...SEX <= -2.204582: 2 (2.7/0.5)

SEX > -2.204582:

:...PULSE <= 0.009931089: 1 (14.1/0.2)

PULSE > 0.009931089:

:...DP <= -0.799774: 1 (4.3/0.3)

DP > -0.799774: 2 (14.7/3.5)

----- Trial 38: -----

Decision tree:

RES > 0.7058755:

:...HBV1 > -0.1472177: 2 (5.9)

: HBV1 <= -0.1472177:

: :...MONO1 <= -1.292171: 2 (12.6/0.7)

: MONO1 > -1.292171:

: :...PULSE > 0.8341221: 2 (9.5)

: PULSE <= 0.8341221:

: :...LYM1 <= -1.111005: 2 (7.8/1.8)

: LYM1 > -1.111005: 1 (11.6/0.2)

RES <= 0.7058755:

:...DM2 > -0.3974532:

:...HCRP1 > -0.2800881: 2 (5.1)

: HCRP1 <= -0.2800881:

: :...DP <= -1.036044: 2 (8.4/0.2)

: DP > -1.036044:

: :...PCT2 > -0.3162744: 1 (4)

: PCT2 <= -0.3162744:

: :...PCT1 <= -0.9715286: 2 (3.5)

: PCT1 > -0.9715286:

: :...RES <= 0.05790384: 1 (9.3/0.8)

: RES > 0.05790384: 2 (5.4/0.8)

DM2 <= -0.3974532:

:...PCT1 > -0.9715286:

:...NOH > 1.78983: 2 (6.1)

: NOH <= 1.78983:

: :...SMK > -1.67839: 1 (66.8/14.3)

: SMK <= -1.67839:

: :...PHD3 > -0.556608: 2 (2.9)

: PHD3 <= -0.556608:

: :...MONO1 <= -1.292171: 2 (6.9/1.7)

: MONO1 > -1.292171: 1 (10.6/0.7)

PCT1 <= -0.9715286:

:...ESR2 <= -0.8700863:

:...HCRP2 > -0.8173213: 2 (22.1/4.2)

: HCRP2 <= -0.8173213:

: :...TEMP > 1.21706: 2 (5.7)

: TEMP <= 1.21706:

: :...NOH > 0.8938254: 1 (2.9)

: NOH <= 0.8938254:

: :...NOH > -0.3605804:

: :...NEUT2 <= -1.104199: 2 (8.4)

: : NEUT2 > -1.104199: 1 (5.9/1.5)

: NOH <= -0.3605804:

: :...NEUT1 > -0.854023: 1 (19.4/3.1)

: NEUT1 <= -0.854023:

: :...SMK <= -1.67839: 2 (4)

: SMK > -1.67839:

: :...NOH <= -0.5397812: 1 (16.6/4.7)

: NOH > -0.5397812: 2 (3.2)

ESR2 > -0.8700863:

:...DP > 0.5390874: 1 (13.7)

DP <= 0.5390874:

:...CVD1 > -0.2025196: 1 (2.8)

CVD1 <= -0.2025196:

:...CRP2 <= -1.275555: 1 (5.5/0.5)

CRP2 > -1.275555:

:...EO1 > -0.6598135: 2 (12.6/1.8)

EO1 <= -0.6598135:

:...AGE <= -0.6266577: 1 (9.3)

AGE > -0.6266577:

:...RES > -0.3740772: 1 (4.6)

RES <= -0.3740772:

:...PULSE <= -0.8731307: 1 (3.7)

PULSE > -0.8731307: 2 (12.3/0.8)

----- Trial 39: -----

Decision tree:

CD2 > -0.3974532:

:...TEMP > 0.1089067: 2 (19.6/1.3)

: TEMP <= 0.1089067:

: :...NOH <= 0.1770221: 1 (11.6/2.3)

: NOH > 0.1770221: 2 (4.9)

CD2 <= -0.3974532:

:...ESR2 > -0.8700863:

:...HCRP2 > -0.8173213:

: :...CRP1 > -0.556608: 2 (4.7/0.2)

: : CRP1 <= -0.556608:

: : :...NEUT1 > -0.854023: 1 (9.3/1.5)

: : NEUT1 <= -0.854023:

: : :...TEMP <= -0.07578556: 1 (19.6/4.6)

: : TEMP > -0.07578556: 2 (21.5/5.4)

: HCRP2 <= -0.8173213:

: :...WBC1 <= -1.189455: 1 (29.3/3.6)

: WBC1 > -1.189455:

: :...CVD1 > -0.2025196: 1 (3)

: CVD1 <= -0.2025196:

: :...PHD3 > -0.556608: 2 (5.3)

: PHD3 <= -0.556608:

: :...MT1 > -0.3220414: 2 (2.8)

: MT1 <= -0.3220414:

: :...PULSE <= 0.304285: 1 (8.4)

: PULSE > 0.304285: 2 (7.9/2.3)

ESR2 <= -0.8700863:

:...CVD1 > -0.2025196: 2 (10.3/2.1)

CVD1 <= -0.2025196:

:...PCT1 > -0.9715286:

:...BRCH3 > -0.2330699: 2 (8/0.8)

: BRCH3 <= -0.2330699:

: :...EO2 > -1.418525:

: :...NOH <= 1.073026: 1 (32/5.1)

: : NOH > 1.073026: 2 (4.8)

: EO2 <= -1.418525:

: :...NEUT2 > -1.104199: 1 (5.7/0.7)

: NEUT2 <= -1.104199:

: :...PULSE <= -1.344097: 1 (2.5)

: PULSE > -1.344097: 2 (14/1.4)

PCT1 <= -0.9715286:

:...TEMP <= -0.8145545: 2 (30.2/3)

TEMP > -0.8145545:

:...NEUT1 > -0.854023:

:...PHD3 > -0.556608: 2 (4/1)

: PHD3 <= -0.556608:

: :...SP <= 0.3272981: 1 (18.9/3.3)

: SP > 0.3272981: 2 (5/0.6)

NEUT1 <= -0.854023:

:...HCRP2 > -0.8173213: 2 (7)

HCRP2 <= -0.8173213:

:...LYM1 > -1.111005: 2 (11.9/2.2)

LYM1 <= -1.111005:

:...MONO1 <= -1.292171: 2 (11/1.6)

MONO1 > -1.292171: 1 (15.7/3.5)

Evaluation on training data (329 cases):

Trial Decision Tree

----- ----------------

Size Errors

0 39 23( 7.0%)

1 23 67(20.4%)

2 28 58(17.6%)

3 30 58(17.6%)

4 24 55(16.7%)

5 26 51(15.5%)

6 36 44(13.4%)

7 26 62(18.8%)

8 35 53(16.1%)

9 27 55(16.7%)

10 39 55(16.7%)

11 25 74(22.5%)

12 29 49(14.9%)

13 26 50(15.2%)

14 26 57(17.3%)

15 33 39(11.9%)

16 33 55(16.7%)

17 36 44(13.4%)

18 27 59(17.9%)

19 29 51(15.5%)

20 29 53(16.1%)

21 27 65(19.8%)

22 34 44(13.4%)

23 24 73(22.2%)

24 34 54(16.4%)

25 31 43(13.1%)

26 30 50(15.2%)

27 29 61(18.5%)

28 34 50(15.2%)

29 26 55(16.7%)

30 30 65(19.8%)

31 33 41(12.5%)

32 26 56(17.0%)

33 33 48(14.6%)

34 31 59(17.9%)

35 32 42(12.8%)

36 31 48(14.6%)

37 20 66(20.1%)

38 33 37(11.2%)

39 28 71(21.6%)

boost 0( 0.0%) <<

(a) (b) <-classified as

---- ----

167 (a): class 1

162 (b): class 2

Attribute usage:

100.00% SMK

100.00% SEX

100.00% NOH

100.00% TEMP

100.00% PULSE

100.00% RES

100.00% SP

100.00% DM2

100.00% CD2

100.00% MT1

100.00% HCRP1

100.00% HCRP2

100.00% ESR2

100.00% LYM1

100.00% LYM2

100.00% PCT1

99.70% AGE

99.70% DP

98.78% PHD3

98.18% CVD1

97.57% CRP2

97.26% PCT2

96.66% EO1

96.66% MONO1

96.05% HTN2

95.74% BRCH3

92.71% ESR1

92.10% NEUT1

85.41% WBC1

78.72% CRP1

77.20% CKD1

62.01% MONO2

53.80% EO2

51.06% WBC2

48.63% HBV1

45.90% NEUT2

Time: 0.4 secs
